# A Genetic Screen Reveals that Synthesis of 1,4-Dihydroxy-2-Naphthoate (DHNA), but Not Full-Length Menaquinone, Is Required for *Listeria monocytogenes* Cytosolic Survival

**DOI:** 10.1128/mBio.00119-17

**Published:** 2017-03-21

**Authors:** Grischa Y. Chen, Courtney E. McDougal, Marc A. D’Antonio, Jonathan L. Portman, John-Demian Sauer

**Affiliations:** a Department of Medical Microbiology and Immunology, University of Wisconsin—Madison, Madison, Wisconsin, USA; b Graduate Group in Infectious Diseases and Immunity, School of Public Health, University of California Berkeley, Berkeley, California, USA; University of Michigan

**Keywords:** *Listeria monocytogenes*, cell autonomous defense, cytosolic pathogen, inflammasome, menaquinone, nutritional immunity, respiration

## Abstract

Through unknown mechanisms, the host cytosol restricts bacterial colonization; therefore, only professional cytosolic pathogens are adapted to colonize this host environment. *Listeria monocytogenes* is a Gram-positive intracellular pathogen that is highly adapted to colonize the cytosol of both phagocytic and nonphagocytic cells. To identify *L. monocytogenes* determinants of cytosolic survival, we designed and executed a novel screen to isolate *L. monocytogenes* mutants with cytosolic survival defects. Multiple mutants identified in the screen were defective for synthesis of menaquinone (MK), an essential molecule in the electron transport chain. Analysis of an extensive set of MK biosynthesis and respiratory chain mutants revealed that cellular respiration was not required for cytosolic survival of *L. monocytogenes* but that, instead, synthesis of 1,4-dihydroxy-2-naphthoate (DHNA), an MK biosynthesis intermediate, was essential. Recent discoveries showed that modulation of the central metabolism of both host and pathogen can influence the outcome of host-pathogen interactions. Our results identify a potentially novel function of the MK biosynthetic intermediate DHNA and specifically highlight how *L. monocytogenes* metabolic adaptations promote cytosolic survival and evasion of host immunity.

## IMPORTANCE

Cytosolic bacterial pathogens, such as *Listeria monocytogenes* and *Francisella tularensis*, are exquisitely evolved to colonize the host cytosol in a variety of cell types. Establishing an intracellular niche shields these pathogens from effectors of humoral immunity, grants access to host nutrients, and is essential for pathogenesis. Through yet-to-be-defined mechanisms, the host cytosol restricts replication of non-cytosol-adapted bacteria, likely through a combination of cell autonomous defenses (CADs) and nutritional immunity. Utilizing a novel genetic screen, we identified determinants of *L. monocytogenes* cytosolic survival and virulence and identified a role for the synthesis of the menaquinone precursor 1,4-dihydroxy-2-naphthoate (DHNA) in cytosolic survival. Together, these data begin to elucidate adaptations that allow cytosolic pathogens to survive in their intracellular niches.

## INTRODUCTION

Despite major advances in understanding how cells detect pathogens in the cytosol (1), little is known about the factors that protect the cytosol from invasion by bacterial pathogens. Likewise, despite a number of studies over the past two decades demonstrating that cells protect their cytosol from bacterial invasion (2–4), how cytosolic pathogens avoid these host defenses and utilize the cytosol as a replication niche remains largely unknown. *Listeria monocytogenes* is a deadly food-borne pathogen (5) that invades the cytosol of a wide variety of cell types and maintains its intracellular niche through coordinated expression of well-characterized virulence factors (6). Maintenance of an intracellular niche is essential for *L. monocytogenes* pathogenesis, and induction of host cell death highly attenuates bacterial virulence (7). We had previously identified a highly conserved protein of unknown function (YvcK) required for survival of *L. monocytogenes* in the macrophage cytosol (8). Bacterial killing in the cytosol resulted in the release of DNA from lysed bacteria, activation of the AIM2 inflammasome, and induction of a programmed cell death process known as pyroptosis (8–11). Pyroptosis attenuates infection by eliminating the replication niche of *L. monocytogenes* (12, 13); as such, cytosolic survival and avoidance of detection by the AIM2 inflammasome are critical for *L. monocytogenes* pathogenesis.

Although numerous determinants of *L. monocytogenes* cytosolic replication have been identified (14–16), few determinants of *L. monocytogenes* cytosolic survival are known (8, 17, 18). Thus, we designed and executed a novel genetic screen to identify *L. monocytogenes* mutants which lyse in the cytosol of macrophages. We identified mutations in genes regulating central metabolism and genes of unknown function critical for *L. monocytogenes* survival. Some genes were selectively required for survival in macrophages but not in other cell types, signifying cell type-specific cytosolic defenses. Unexpectedly, through an as-yet-undefined mechanism, a subset of mutants that lyse in the cytosol still avoided inflammasome activation. Despite this, all mutants identified in the screen were attenuated in a murine model of listeriosis. Next we investigated the function of menaquinone (MK) in cytosolic survival. We found that MK’s canonical functions in cellular respiration and the electron transport chain (ETC) were not critical for *L. monocytogenes* cytosolic survival. Instead, synthesis of the MK biosynthetic intermediate 1,4-dihydroxy-2-naphthoate (DHNA), but not of fully functional, isoprenylated menaquinone, was required for *L. monocytogenes* cytosolic survival. Taking the data together, our genetic screen uncovered factors required for *L. monocytogenes* survival in the host cytosol and evasion of the innate immune system and ultimately revealed a novel, ETC-independent function for DHNA. Additionally, these results add to the growing body of literature demonstrating that central metabolism plays a key role during host-pathogen interactions. Not only do host cells monitor and modulate their metabolism to sense and respond to pathogens (19), but cytosolic pathogens must also modulate their metabolism to avoid detection and/or killing by hosts.

## RESULTS

### Identification of genes required for cytosolic survival within macrophages.

To explicitly identify genes required for cytosolic survival of *L. monocytogenes*, we designed and executed a novel genetic screen to identify mutants of *L. monocytogenes* that lyse in the cytosol of host cells. Bacteriolysis of *L. monocytogenes* at the population level is indirectly measured through delivery of a luciferase-based reporter plasmid (pBHE573) (8) to the host cytosol during infection. Luciferase expression occurs only if the reporter translocates from the bacteria to the host cytosol since the luciferase gene is transcribed from a cytomegalovirus (CMV) promoter (see Fig. S1A in the supplemental material). We performed a nonsaturating screen using approximately 6,500 independent *L. monocytogenes* transposon mutants carrying pBHE573 and monitored for bacteriolysis (Fig. S1B). Type I interferon receptor-deficient immortalized macrophages (iIFNAR^−/−^) were used to avoid type I interferon-mediated suppression of translation (20), which would impact luciferase expression. Following secondary screening, we prioritized isolates which induced 2-fold or greater increases in luciferase expression compared to wild-type *L. monocytogenes*.

10.1128/mBio.00119-17.1 FIG S1  Screening for *L. monocytogenes* transposon mutants with intracellular survival defects in macrophages. (A) *L. monocytogenes* mutants carrying bacteriolysis reporter pBHE573 that can neither access the host cytosol nor avoid bacteriolysis in the host cytosol induce low levels of luciferase production in the host. However, *L. monocytogenes* mutants with survival defects transfer pBHE573 to the host cytosol and induce high levels of luciferase production. (B) Approximately 6,500 random transposon mutants were tested for increased bacteriolysis within macrophages. Data are normalized to wild-type levels of bacteriolysis. Download FIG S1, EPS file, 0.5 MB. Copyright © 2017 Chen et al. 2017 Chen et al. This content is distributed under the terms of the Creative Commons Attribution 4.0 International license.

Six unique isolates possessed single transposon insertions within the coding region of six different genes (see Table S1 in the supplemental material). *nrdD* encodes an anaerobic nucleotide reductase required for production of nucleotides during anaerobic growth of *L. monocytogenes* (21). *pdhC* encodes the E2 subunit of pyruvate dehydrogenase, which converts pyruvate to acetyl-coenzyme A (acetyl-CoA) while generating NADH (22). *menF* and *menD* encode enzymes for the first two dedicated steps in the biosynthesis of MK, a membrane-bound molecule which shuttles electrons between different complexes of the election transport chain (23). Finally, two genes of unknown function were identified in this genetic screen: *lmo1602* and *yvcJ*. *lmo1602* appears to be a sigma B-regulated gene associated with stress responses (24, 25), whereas *yvcJ* is cotranscribed with *yvcK* (26), a gene previously found to be required for cytosolic survival of *L. monocytogenes* (8).

10.1128/mBio.00119-17.8 TABLE S1  Genes identified for intracellular survival of *L. monocytogenes*. Download TABLE S1, DOCX file, 0.05 MB. Copyright © 2017 Chen et al. 2017 Chen et al. This content is distributed under the terms of the Creative Commons Attribution 4.0 International license.

**FIG 1:**
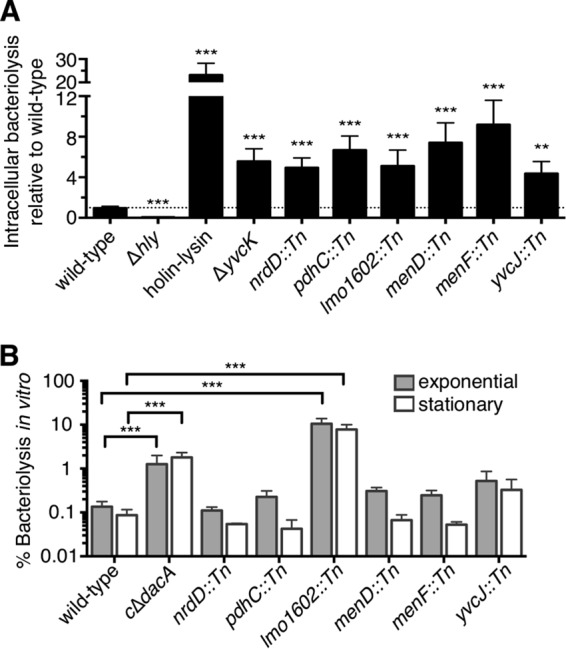
Identification of genes required for cytosolic survival of *L. monocytogenes* in macrophages. (A) Cleanly transduced *L. monocytogenes* bacteriolysis mutants (MOI of 10) were tested for bacteriolysis in *iIFNAR*^*−/−*^ macrophages over a 6-h infection. All data are normalized to wild-type levels of bacteriolysis and presented as means ± standard errors of the mean (SEM) of results from five independent experiments. (B) Cleanly transduced *L. monocytogenes* bacteriolysis mutants were grown to the exponential or stationary phase in BHI media at 37°C and then examined for *in vitro* lysis. β-Galactosidase activities of supernatants are normalized to β-galactosidase activities of 100% lysed cultures, and data are presented as mean percent ± SEM of results from four independent experiments.

To exclude the possibility of secondary mutations, we transduced the transposon mutations into clean wild-type backgrounds and reexamined these strains for survival in macrophages (Fig. 1A). Holin-lysin, a *L. monocytogenes* strain expressing inducible bacteriophage holin and lysin proteins, and Δ*yvcK* mutants were used as positive bacteriolysis controls (8). Δ*hly* mutants which cannot escape the host vacuole (27) were used as negative controls (8, 18). *L. monocytogenes* bacteriolysis mutants displayed bacteriolysis that was 4-fold to 8-fold higher than that seen with the wild-type strain, results similar to those obtained with Δ*yvcK* mutants (Fig. 1A). Importantly, while the mutants that we identified lysed more frequently than the wild-type strain, lysis of these strains was still a relatively rare event compared to lysis of the ~100% lysis control, holin-lysin. We also constructed markerless deletions and genetic complements for every gene identified in the genetic screen and tested them for survival in macrophages (Fig. S2A). With the exception of a Δ*pdhC* strain which we were unable to construct, the deletion strains reliably phenocopied their transposon mutant counterparts. Additionally, complementation of clean deletions and transposon mutants of the majority of mutants was successful, with the exception of Δ*nrdD* and Δ*yvcJ* mutants. Furthermore, to test whether mutants undergo complete bacteriolysis and not simply plasmid secretion, we created a chromosomal CMV-luciferase reporter and assayed *L. monocytogenes* mutants for intracellular survival. *pdhC*::*Tn*, *menD*::*Tn*, *menF*::*Tn*, and *yvcJ*::*Tn* mutants underwent complete bacteriolysis (Fig. S2B). The level of luciferase production from the chromosomal lysis reporter was significantly reduced compared to that seen in the plasmid-based assay, and as such, both the wild-type strain and the mutants demonstrated low levels of lysis that were below the limit of detection.

10.1128/mBio.00119-17.2 FIG S2  Bacteriolysis mutants identified in the genetic screen undergo complete bacteriolysis, inside macrophages. (A) Genetic complements of clean deletions and transposon mutants (MOI of 10) were tested for bacteriolysis in *iIFNAR*^*−/−*^ macrophages using the bacteriolysis assay. Data are normalized to wild-type levels of bacteriolysis and presented as means ± SEM of results from six independent experiments. NT, not tested. (B) Bacteriolysis mutants carrying chromosomal bacteriolysis reporter pYL56 (MOI of 10) were tested for bacteriolysis in *iIFNAR*^*−/−*^ macrophages 6 h postinfection. Ampicillin (Ap; 1 mg/ml) was added to the wild-type infection as a positive control. All data are presented as mean fluorescence ± SEM from six independent experiments. Download FIG S2, EPS file, 1.2 MB. Copyright © 2017 Chen et al. 2017 Chen et al. This content is distributed under the terms of the Creative Commons Attribution 4.0 International license.

Using an *in vitro* bacteriolysis assay (28), we also examined each mutant for bacteriolysis during exponential-phase and stationary-phase growth in broth culture (Fig. 1B). As a positive control, we used a conditional mutant of diadenylate cyclase (cΔ*dacA*), a gene essential for growth of *L. monocytogenes* in nutrient-rich media and for survival *in vitro* (28, 29). The *lmo1602*::*Tn* mutant was the only mutant which significantly lysed in broth culture, suggesting that this gene is essential for survival *in vitro*. These data highlight the idea that other genes identified in our genetic screen are required for survival in the macrophage cytosolic environment but not during extracellular replication.

Salmonella enterica subsp. Typhimurium Δ*sifA* mutants escape to the host cytosol and replicate in epithelial cells but are killed upon entry into the macrophage cytosol, suggesting the presence of both cell type-specific cell autonomous defenses (CADs) and bacterial survival mechanisms (3). We next asked whether the cytosolic survival defects of our mutants were macrophage specific. Using the plasmid-based bacteriolysis assay, we assessed survival of each of our mutants in epithelial (Caco-2) cells and fibroblasts (BHKs) (Fig. S3A and B). Only the *yvcJ*::*Tn* mutant was significantly impaired for survival in all cell types, suggesting that YvcJ is generally required for cytosolic survival. *nrdD*::*Tn*, *pdhC*::*Tn*, *menD*::*Tn*, and *menF*::*Tn* mutants were significantly impaired for survival in macrophages and Caco-2 cells but not BHKs. Surprisingly, given the *in vitro* and macrophage bacteriolysis phenotypes, *lmo1602*::*Tn* mutants were not impaired for survival in either Caco-2 cells or BHKs. These observations of cell type-specific bacteriolysis are consistent with the idea of specific CADs and/or nutritional immunity in the cytosol of different cell types.

10.1128/mBio.00119-17.3 FIG S3  Bacteriolysis mutants differentially lyse in Caco-2 and BHKs. (A and B) Bacteriolysis mutants were tested for bacteriolysis in Caco-2 cells (A) or BHKs (B) (MOI of 64 and 100, respectively) using the bacteriolysis assay. Data are normalized to wild-type levels of bacteriolysis and presented as means ± SEM of results from six independent experiments. Download FIG S3, EPS file, 0.8 MB. Copyright © 2017 Chen et al. 2017 Chen et al. This content is distributed under the terms of the Creative Commons Attribution 4.0 International license.

### Genes required for cytosolic survival are also required for virulence.

**FIG 2:**
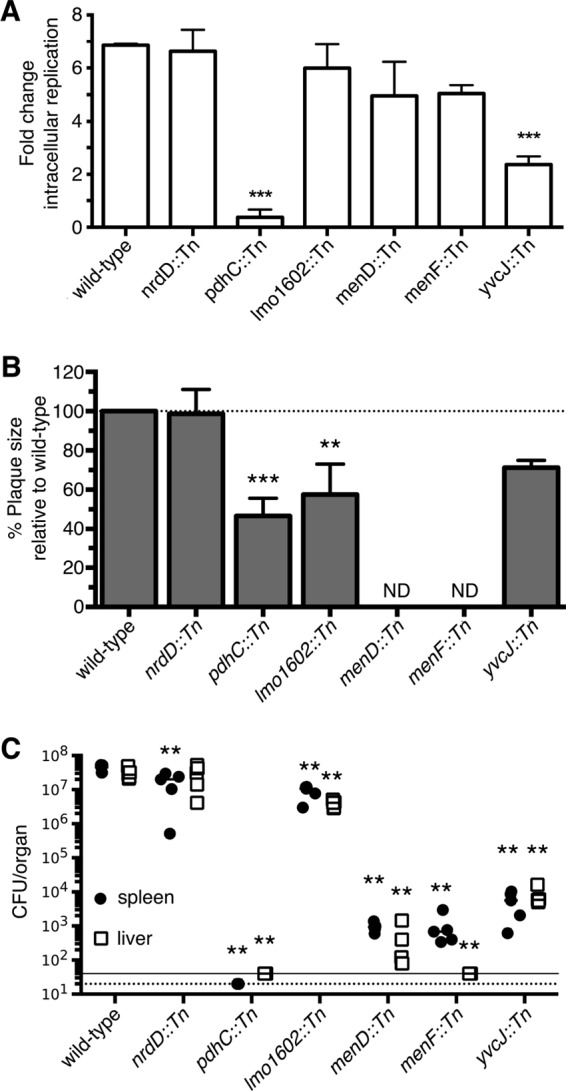
Genes required for cytosolic survival are also required for virulence. (A) Bacteriolysis mutants (MOI of 0.2) were grown in bone marrow-derived macrophages (BMDMs) and then enumerated for CFU at 2 and 5 h postinfection. Data are calculated as fold change between 2 and 5 h and representative of four independent experiments. (B) *L. monocytogenes* bacteriolysis mutants (MOI of 0.5) were examined for plaque formation in L2 fibroblasts 6 days postinfection. Data are normalized to wild-type plaque size and represent means ± SEM of results from three independent experiments. ND, not detected. (C) Bacterial burdens (CFU) from the spleen (●) and liver (□) were enumerated at 48 h postinfection. Data are representative of results from two biological replicates. The horizontal dotted and solid lines denote the limits of detection for the spleen and livers, respectively. Mann-Whitney statistical analysis was performed to measure statistical significance in comparison to wild-type results.

On the basis of the holin-lysin data that suggest that lysis of the mutants is a rare event, we asked whether cytosolic survival correlates with intracellular replication. We first assessed intracellular replication in wild-type C57BL/6 bone marrow-derived macrophages (BMDMs) (Fig. 2A and Fig. S4A). *pdhC*::*Tn* mutants are gradually cleared over time, whereas *yvcJ*::*Tn* mutants displayed partial replication defects in macrophages, similarly to previous observations for Δ*yvcK* mutants (8). Other mutants did not display replication defects within macrophages, suggesting that bacteriolysis is incomplete and that intracellular bacteriolysis does not strictly correlate with intracellular replication. Although MK mutants could not grow in minimal media lacking MK, MK-deficient mutants grown in MK-limited media displayed a small but reproducible invasion defect, potentially due to decreased internalization or decreased phagosomal survival. In addition, and consistent with previous results (30, 31), MK-starved MK-deficient mutants were impaired for intracellular replication (Fig. S4B), suggesting that carryover of MK from brain heart infusion can support intracellular replication over the course of infection. Furthermore, while individual bacteriolysis mutants displayed differential growth phenotypes in a variety of cell types, the ability to replicate intracellularly did not correlate with cell type-specific lysis (Fig. S4C to E).

10.1128/mBio.00119-17.4 FIG S4  Intracellular replication of bacteriolysis in different cell types. (A) Bacteriolysis mutants (MOI of 0.2) were examined for intracellular replication in BMDMs. This panel depicts the data from the experiment in Fig. 2A but displayed as CFU at 0.5, 2, 5, and 8 h postinfection. (B) Δ*menD* and *ΔmenF* strains were grown in minimal media plus 25 ng/ml MK overnight. Infected BMDMs at a starting MOI of 0.2 were then enumerated for CFU at 2 and 5 h postinfection. MK (50 μg/ml) was added exogenously to infected cells to complement growth of MK-deficient strains. Data are calculated as fold change between 2 and 5 h and representative of results from three independent experiments. (C to E) Bacteriolysis mutants were grown in primary *IFNAR*^*−/−*^ macrophages (MOI of 0.2) (C), Caco-2 cells (MOI of 5) (D), and BHKs (MOI of 5) (E) and then enumerated for CFU at 2 and 5 h postinfection. Data are calculated as fold change between 2 and 5 h and representative of results of two independent experiments. (B to E) The panels on the right depict the same data but graphed as CFU at 0.5, 2, 5, and 8 h postinfection. Download FIG S4, TIF file, 1.8 MB. Copyright © 2017 Chen et al. 2017 Chen et al. This content is distributed under the terms of the Creative Commons Attribution 4.0 International license.

Despite the lack of correlation between bacteriolysis and intracellular replication, we hypothesized that cytosolic survival was essential for virulence. To assess virulence *ex vivo*, we tested these mutants for plaque formation within L2 fibroblasts (32) (Fig. 2B). *menD*::*Tn* and *menF*::*Tn* mutants did not form visible plaques even after 6 days of infection. *lmo1602*::*Tn* and *yvcJ*::*Tn* mutants had intermediate virulence phenotypes, while *nrdD*::*Tn* mutants made plaques similarly to wild-type *L. monocytogenes*. Strikingly, *pdhC*::*Tn* mutants were capable of forming intermediate-sized plaques despite being cleared in macrophages, consistent with the *pdhC*::*Tn* mutant’s minimal bacteriolysis phenotype in fibroblasts (Fig. S3B).

Finally, we examined virulence *in vivo* using a murine acute infection model of listeriosis (Fig. 2C). Most mutants were significantly attenuated for virulence in both the spleen and the liver. Interestingly, *nrdD*::*Tn* mutants, which should be unable to grow anaerobically (21), were attenuated only moderately in the spleen and were not attenuated in the liver, suggesting that these particular niches for *L. monocytogenes* are not completely devoid of oxygen. The most attenuated strains were *pdhC*::*Tn*, *menD*::*Tn*, *menF*::*Tn*, and *yvcJ*::*Tn* mutants. Together, these data show that, independently of cell type-specific survival defects, genes required for cytosolic survival are essential for virulence *in vivo*.

### Bacteriolysis mutants differentially activate the inflammasome.

**FIG 3:**
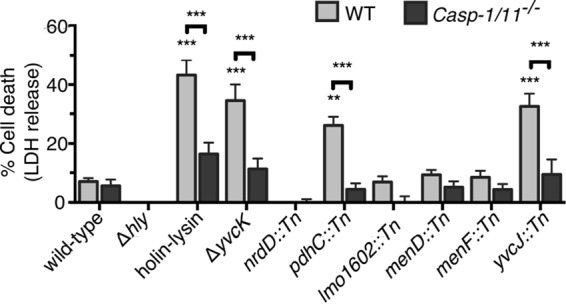
Genes required for cytosolic survival differentially activate the inflammasome. Lactate dehydrogenase assays were performed by infecting wild-type (WT) or caspase-1- and caspase-11-deficient (*Casp-1* and *Casp-11*^*−/−*^) BMDMs with *L. monocytogenes* bacteriolysis mutants (MOI of 1). Data are presented as means ± SEM of results from three independent experiments. LDH, lactate dehydrogenase.

Previous studies discovered that *L. monocytogenes* and *Francisella* species mutants hyperactivated the inflammasome as a consequence of bacteriolysis in the host cytosol (8, 10); hence, we hypothesized that our bacteriolysis mutants would also induce pyroptosis in macrophages. Holin-lysin and Δ*yvcK* strains were used as positive controls, since lysis of these strains triggers the AIM2 inflammasome (8). The Δ*hly* strain induces undetectable levels of cell death because it cannot access the cytosol to be recognized by AIM2 (8). Infections with *pdhC*::*Tn* and *yvcJ*::*Tn* mutants induced significantly higher levels of caspase-1- and AIM2-dependent cell death in macrophages in comparison to wild-type results as expected (Fig. 3 and Fig. S5A). Like that seen with the Δ*yvcK* mutants (8), *yvcJ*::*Tn* mutant intracellular replication was partially caspase-1 dependent, as indicated by a moderate rescue in intracellular replication in caspase-1-deficient macrophages (Fig. S5B). Unlike that seen with the *ΔyvcK* and *yvcJ*::*Tn* mutants, *pdhC*::*Tn* mutant intracellular replication was not rescued in caspase-1-deficient macrophages, suggesting that the intracellular growth defect of these mutants is independent of induction of host cell death (Fig. S5C). Additionally, despite delivering plasmid and chromosomal DNA to the cytosol (Fig. 1A and Fig. S2C), infections with the other bacteriolysis mutants induced cell death at levels indistinguishable from those seen with the wild-type strain (Fig. 3). These mutants also did not induce caspase-3/caspase-7 (caspase-3/7)-dependent apoptosis in macrophages as an alternative to pyroptosis (Fig. S5D). In contrast to previous reports demonstrating a consistent link between bacteriolysis and inflammasome activation (8, 10), our data suggest that cytosolic bacteriolysis alone may be insufficient to trigger the inflammasome or that *L. monocytogenes* possesses additional strategies to avoid inflammasome activation.

10.1128/mBio.00119-17.5 FIG S5  Bacteriolysis mutants do not induce apoptosis. (A) Lactate dehydrogenase assays were performed infecting wild-type (WT) and *AIM2*^*−/−*^ BMDMs with bacteriolysis strains (MOI of 1). Data are presented ± SEM of results from two independent experiments. (B and C) *yvcJ*::*Tn* (B) and *pdhC*::*Tn* (C) strains (MOI of 0.2) were grown in BMDMs and then enumerated for CFU at 2 and 5 h postinfection. Data are calculated as fold change between 2 and 5 h and representative of results of two independent experiments. The panels on the right depict the same data but graphed as CFU at 0.5, 2, 5, and 8 h postinfection (D) *iIFNAR*^*−/−*^ macrophages infected with bacteriolysis mutants at a MOI of 10 and measured for caspase-3/7 activation 6 h postinfection. Data are presented as means ± SEM of results from three independent experiments. NS, not significant. Download FIG S5, EPS file, 1.8 MB. Copyright © 2017 Chen et al. 2017 Chen et al. This content is distributed under the terms of the Creative Commons Attribution 4.0 International license.

### Menaquinone biosynthesis is required for intracellular survival of *L. monocytogenes*.

**FIG 4:**
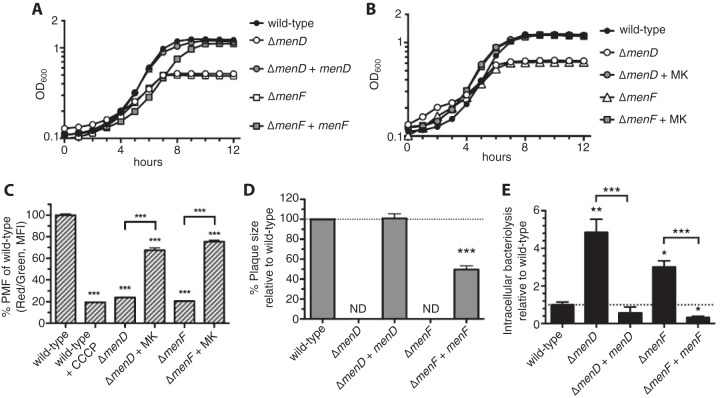
Δ*menD* and Δ*menF* strains are deficient for generation of the membrane potential and intracellular survival. (A and B) Genetic (A) or chemical (B) complementation of Δ*menD* and Δ*menF* strains grown in aerated cultures in BHI media at 37°C and monitored at OD_600_. Data are representative of results from three independent experiments. (C) Δ*menD* and *ΔmenF* cultures grown to exponential phase in BHI media at 37°C were examined for membrane potential generation. Data are normalized to the wild-type red/green fluorescence ratios and represented as mean percent ± SEM of results from three independent experiments. MFI, median fluorescence intensity; PMF, proton motive force. (D) *ΔmenD* and *ΔmenF* strains (MOI of 0.5) were examined for plaque formation in L2 fibroblasts 6 days postinfection. Data are normalized to wild-type plaque size and represent mean percent ± SEM of results from three independent experiments. ND, not detected. (E) *ΔmenD* and *ΔmenF* strains (MOI of 10) were tested for bacteriolysis in *iIFNAR*^*−/−*^ macrophages over 6 h. All data are normalized to wild-type levels of bacteriolysis and presented as means ± SEM of results from four independent experiments.

In addition to the original 12 bacteriolysis mutants, we isolated a strain with multiple mutations in its chromosome. In this mutant, deletion of shikimate biosynthesis genes (*aroED*) was responsible for the bacteriolysis phenotype (data not shown) and led to MK auxotrophy since this pathway produces a precursor for MK (30) (see Fig. 6A). This, combined with our isolation of *menD* and *menF* mutants, prompted us to examine the role of MK in *L. monocytogenes* cytosolic survival. Consistent with our data from the transposon mutants as well as with previously published results (30, 31), the Δ*menD* and Δ*menF* strains can neither efficiently replicate aerobically nor generate a robust membrane potential (Fig. 4A to C). Additionally, these mutants were significantly attenuated for virulence and intracellular survival in macrophages (Fig. 4D and E).

To understand what host defenses may induce intracellular bacteriolysis, we examined these mutants for sensitivity to a variety of stresses. MK auxotrophs were not sensitive to cell wall- or cell membrane-targeting antimicrobials (Fig. S6A and B). Although sensitivity to these antimicrobials could be masked by trace amounts of MK in the brain heart infusion (BHI) media, MK auxotrophs were still hypersensitive to oxidative stress in BHI cultures (Fig. S6C). We next examined cellular reactive oxygen species (ROS) production in macrophages infected with the wild-type strain or the Δ*menD* mutant but did not detect increases in ROS production during infection (Fig. S6D). Though ROS scavenging with N-acetylcysteine (NAC) did partially lower ROS production in menadione-induced macrophages (positive control), NAC treatment did not rescue MK mutants from bacteriolysis, suggesting that cytosolic ROS is not responsible for the intracellular survival defects of MK-deficient mutants, though additional evidence may be required (Fig. S6D and E). These data leave open the possibility that another unknown cytosolic stress(es) or host defense may be responsible for lysis of MK-deficient *L. monocytogenes* in macrophages.

10.1128/mBio.00119-17.6 FIG S6  MK-deficient strains are sensitive to ROS *in vitro* but not *ex vivo*. (A to C) The *menD*::*Tn* and *menF*::*Tn* strains were tested for sensitivity to various concentrations of ceftriaxone (CRO), ampicillin (AMP), bacitracin (BAC), and daptomycin (DAP) (A); lysozyme and LL-37 (B); and hydrogen peroxide (C) *in vitro* to calculate MICs. Data represent median MICs of results from three or more independent experiments. (D) *iIFNAR*^*−/−*^ macrophages were treated with 100 µM menadione for 1 h or infected with the wild-type strain or the Δ*menD* strain at an MOI of 10 for 6 h. Cells were treated with or without 1mM N-acetylcysteine (NAC) throughout the assay and were then examined for ROS production. (E) The Δ*menD* strain (MOI of 10) was examined for bacteriolysis in *iIFNAR*^*−/−*^ macrophages 6 h postinfection. NAC (1 mM) was added to infections to scavenge ROS. Data are normalized to wild-type levels of bacteriolysis and presented as means ± SEM of results from four independent experiments. NS, not significant. Download FIG S6, EPS file, 0.7 MB. Copyright © 2017 Chen et al. 2017 Chen et al. This content is distributed under the terms of the Creative Commons Attribution 4.0 International license.

### Loss of the electron transport chain does not fully account for MK mutant survival defects.

**FIG 5:**
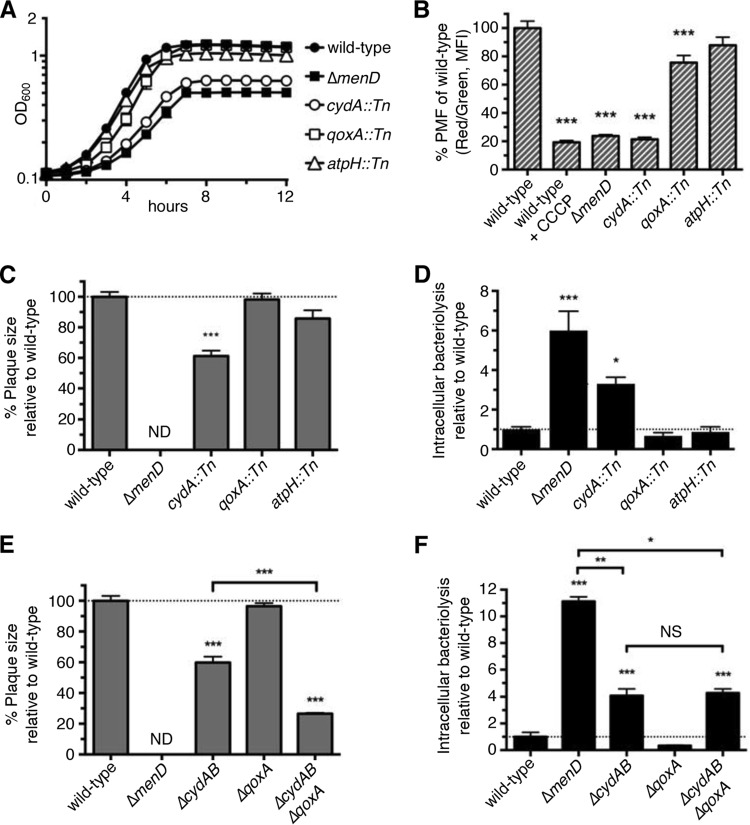
The electron transport chain plays a minor role in survival of *L. monocytogenes* within macrophages. (A) Δ*menD*, *cydA*::*Tn*, *qoxA*::*Tn*, and *atpH*::*Tn* strains were grown in aerated BHI cultures at 37°C and monitored for OD_600_. Data are representative of results from three independent experiments. (B) Δ*menD*, *cydA*::*Tn*, *qoxA*::*Tn* and *atpH*::*Tn* strains grown to exponential phase in BHI media at 37°C were examined for membrane potential generation. Data are normalized to the wild-type red/green fluorescence ratios and presented as mean percent ± SEM of results from three independent experiments. (C and E) Δ*menD*, *cydA*::*Tn*, *qoxA*::*Tn*, and *atpH*::*Tn* strains (C) or Δ*menD*, *ΔcydAB*, *ΔqoxA*, and Δ*cydAB/ΔqoxA* strains (E) (MOI of 0.5) were examined for plaque formation in L2 fibroblasts 6 days postinfection. Data are normalized to wild-type plaque size and represent means ± SEM of results from three independent experiments. ND, not detected. (D and F) Δ*menD*, *cydA*::*Tn*, *qoxA*::*Tn*, and *atpH*::*Tn* strains (D) or Δ*menD*, *ΔcydAB*, *ΔqoxA*, and Δ*cydAB/ΔqoxA* strains (F) (MOI of 10) were tested for bacteriolysis in *iIFNAR*^*−/−*^ macrophages using the bacteriolysis assay. Data are normalized to wild-type levels of bacteriolysis and presented as means ± SEM of results from five independent experiments.

Loss of MK biosynthesis in *L. monocytogenes* disrupts the electron transport chain (ETC) and prevents *L. monocytogenes* from producing a robust membrane potential (Fig. 4C). To investigate whether the ETC is required for intracellular survival of *L. monocytogenes*, we characterized transposon mutants with mutations in *cydA*, *qoxA*, and *atpH* (genes encoding ETC components cytochrome *bd* oxidase, cytochrome aa_3_ oxidase, and ATP synthase, respectively) (33). Similarly to MK-deficient mutants, the *cydA*::*Tn* mutant was deficient for generating a membrane potential and displayed *in vitro* aerobic growth defects (Fig. 5A and B). *qoxA*::*Tn* mutants were partially defective with respect to their ability to generate a membrane potential, whereas *atpH*::*Tn* mutants produced a robust membrane potential similar to that seen with the wild-type strain and grew to wild-type levels *in vitro*. No visible growth of the *atpH*::*Tn* mutant was observed under anoxic conditions, likely because ATP synthase is essential for generating a membrane potential during fermentative growth (34) (Fig. S7A). Interestingly, *cydA*::*Tn* mutants did not have a growth defect within macrophages but made significantly smaller plaques (Fig. 5C and Fig. S7B). In contrast, *qoxA*::*Tn* and *atpH*::*Tn* mutants also replicated in macrophages but formed plaques similar to those seen with the wild-type strain, suggesting that neither the cytochrome aa_3_ oxidase nor the ATP synthase of *L. monocytogenes* is important for virulence *ex vivo* (Fig. 5C and Fig. S7B). The *atpH*::*Tn* mutant displayed an initial invasion defect in macrophages (Fig. S7B), though this was likely be due to poorer growth in statically grown overnight cultures. Next we examined ETC complex mutants for intracellular survival. *cydA*::*Tn* mutants, but not *qoxA*::*Tn* or *atpH*::*Tn* mutants, lysed in the cytosol of macrophages, albeit not to Δ*menD* levels (Fig. 5D), suggesting that the ETC or ability to generate a membrane potential may contribute, at least partially, to *L. monocytogenes* intracellular survival.

10.1128/mBio.00119-17.7 FIG S7  Electron transport chain mutations are not impaired for intracellular replication. (A) The *atpH*::*Tn* strain was grown on BHI plates under oxic and anoxic conditions. (B) *cydA*::*Tn*, *qoxA*::*Tn*, and *atpH*::*Tn* strains (MOI of 0.2) were grown in BMDMs and enumerated for CFU at 2 and 5 h postinfection. Data are calculated as fold change between 2 and 5 h and representative of results from three independent experiments. The panel on the right depicts the same data but graphed as CFU at 0.5, 2, 5, and 8 h postinfection. Download FIG S7, EPS file, 1 MB. Copyright © 2017 Chen et al. 2017 Chen et al. This content is distributed under the terms of the Creative Commons Attribution 4.0 International license.

Since *cydA*::*Tn* mutants did not phenocopy Δ*menD* mutants for intracellular survival, we hypothesized that disruption of both cytochrome oxidases might be necessary to completely abolish membrane potential; hence, we generated a double cytochrome oxidase mutant, the Δ*cydAB*/Δ*qoxA* strain. This mutant was more attenuated for virulence than either single cytochrome oxidase mutant (Fig. 5E), suggesting at least partial redundancy of the two cytochrome oxidases in *L. monocytogenes*. Unexpectedly, the double Δ*cydAB*/Δ*qoxA* mutants lysed at the same level as single Δ*cydAB* mutants (Fig. 5F). Although the cytochrome oxidase mutants exhibited moderate intracellular survival defects, our data suggest that noncanonical functions of MK, independently of MK-dependent functions in the ETC, protect *L. monocytogenes* from cytosolic bacteriolysis.

### Biosynthesis of DHNA is required for cytosolic survival of *L. monocytogenes* in macrophages.

**FIG 6:**
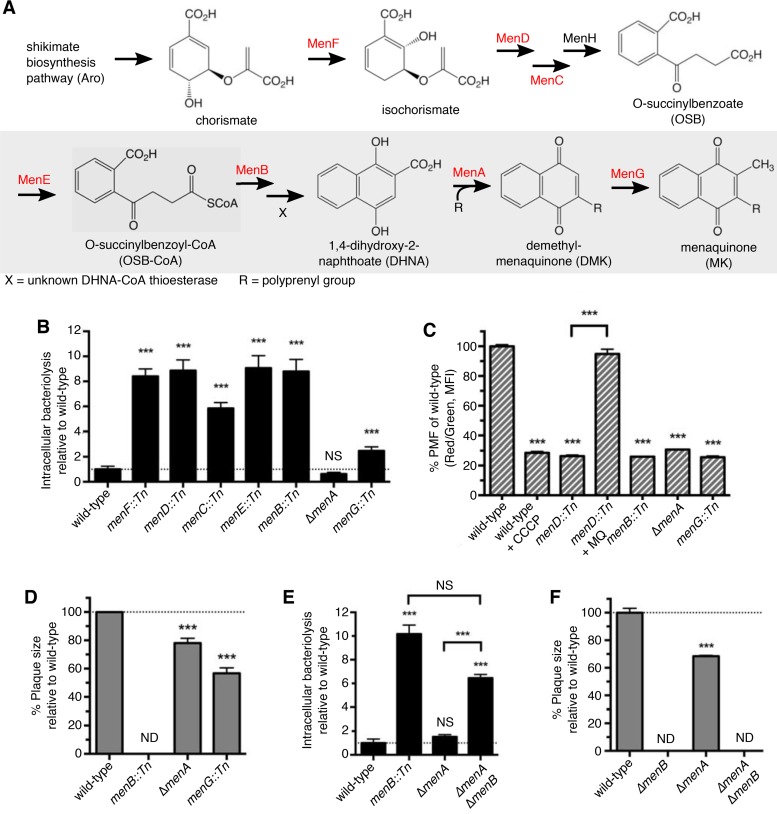
MK biosynthesis intermediates are required for cytosolic survival of *L. monocytogenes*. (A) Major intermediates and enzymes present in the MK biosynthesis pathway of *L. monocytogenes* according to the KEGG database (http://www.genome.jp/kegg/). Specific *men* mutants examined in this study are highlighted in red. (B and E) MK biosynthesis mutants (B) or Δ*menB*, *ΔmenA*, and Δ*menB/ΔmenA* strains (E) (MOI of 10) were tested for bacteriolysis in *iIFNAR*^*−/−*^ macrophages using the bacteriolysis assay. Data are normalized to wild-type levels of bacteriolysis and presented as means ± SEM of results from four to six independent experiments. (C) The *menD*::*Tn*, *menB*::*Tn*, *ΔmenA*, and *menG*::*Tn* strains were grown to exponential phase in BHI media at 37°C of stained and measured for membrane potential generation. Data are normalized to wild-type red/green fluorescence ratios and presented as mean percent ± SEM of results from three independent experiments. (D and F) The *menB*::*Tn*, *ΔmenA*, and *menG*::*Tn* strains (D) or Δ*menB*, *ΔmenA*, and Δ*menB/ΔmenA* strains (MOI of 0.5) were examined for plaque formation in L2 fibroblasts at 6 days postinfection. Data are normalized to wild-type plaque size and represent means ± SEM of results from three independent experiments.

The results described above led us to reexamine the role of MK biosynthesis in intracellular survival of *L. monocytogenes*. MK is synthesized from chorismate, the end product of the shikimate biosynthesis pathway, by eight well-characterized enzymes encoded by the *men* genes (23) (Fig. 6A). Following conversion of o-succinylbenzoyl-CoA (OSB-CoA) to 1,4-dihydroxy-2-naphthoyl-CoA (DHNA-CoA) by MenB, an unknown DHNA-CoA thioesterase is predicted to convert DHNA-CoA to 1,4-dihydroxy-2-naphthoate (DHNA) (35). MenA and MenG are responsible for the final two steps in MK biosynthesis by addition of the polyprenyl side chain and methylation of the naphthoquinone ring, respectively (Fig. 6A).

To investigate how MK biosynthesis drives intracellular survival of *L. monocytogenes* in macrophages, we obtained transposon mutants (from an unpublished genetic screen that identified small-colony variants) with mutations of every gene encoding enzymes in the MK biosynthesis pathway, with the exception of *menH* and the unknown thioesterase gene. Mutations in genes *menF* through *menB*, which are responsible for the first six steps in MK biosynthesis, led to survival defects of *L. monocytogenes* in macrophages, confirming the findings of our screen (Fig. 6B). Remarkably, the loss of DHNA polyprenyltransferase (MenA) did not lead to intracellular bacteriolysis of *L. monocytogenes*. The *menG*::*Tn* mutant, with a disruption in demethylmenaquinone (DMK) methyltransferase (MenG), displayed only a minor bacteriolysis phenotype, reminiscent of the Δ*cydAB* mutant results. These data suggest that synthesis of DHNA, but not menaquinone, is required for intracellular survival of *L. monocytogenes*. Additionally, all of the MK biosynthesis mutants, including the Δ*menA* mutant, were sensitive to oxidative stress *in vitro* (Fig. S6C), further suggesting that cytosolic ROS is not responsible for bacteriolysis of these mutants. Importantly, mutations in *menB*, *menA*, or *menG* abolish the ability of *L. monocytogenes* to generate a membrane potential (Fig. 6C), highlighting, moreover, that the ETC/membrane potential plays a minor (if any) role in intracellular survival. Curiously, these data also demonstrate that, unlike *Staphylococcus aureus* (36), *L. monocytogenes* cannot use demethylmenaquinone (DMK) in the electron transport since the *menG*::*Tn* strain, which should accumulate DMK, does not respire (Fig. 6C). Δ*menA* and *menG*::*Tn* mutants were impaired for plaque formation similarly to the Δ*cydAB* mutant, though not to the extent seen with other *men* mutants, further suggesting that, in addition to the function of menaquinone for respiration and generation of a membrane potential, biosynthesis of the DHNA is critical for *L. monocytogenes* virulence (Fig. 6D). Finally, to pinpoint the exact step of MK biosynthesis critical for cytosolic survival, we generated a double deletion strain (mutant Δ*menA*/Δ*menB*) and tested it for bacteriolysis in macrophages. *ΔmenA*/*ΔmenB* mutants phenocopied a single Δ*menB* mutant, lysing significantly in macrophages and being drastically attenuated for virulence in a plaquing assay (Fig. 6E and F). Taken together, our data support the model that DHNA, or a derivative of it, is a critical virulence determinant independently of its role in the synthesis of MK for the ETC. Additionally, synthesis of DHNA, through a yet-to-be-defined mechanism, prevents cytosolic lysis of *L. monocytogenes* in macrophages.

## DISCUSSION

How cytosolic pathogens avoid killing in the highly restrictive host cytosol is unknown. Using a novel genetic screen, we identified factors required for cytosolic survival of *L. monocytogenes* inside the host. Our approach uncovered genes involved in central metabolism and genes of unknown function required for the cytosolic survival and, ultimately, virulence of *L. monocytogenes* (see Table S1 in the supplemental material). Our data suggest that *L. monocytogenes* may have distinct strategies to overcome different nutritional limitations, stresses, and/or active CADs in different cytosolic environments (Fig. 1A; see also Fig. S3A and B in the supplemental material). Finally, we demonstrated that synthesis of the MK biosynthesis intermediate DHNA, but not of full-length isoprenylated MK, is essential for *L. monocytogenes* cytosolic survival in macrophages (Fig. 6B and E). Our findings identify some critical metabolic pathways for survival and immune evasion in the host cytosol. Finally, these findings are consistent with (and add to) recent studies demonstrating the intricate connection between pathogen and host metabolism in the context of pathogenesis and innate immune regulation.

Although the minimal amount of DNA required to trigger the AIM2 inflammasome is unknown, studies of *Francisella* have shown that inflammasome components can be recruited to singly lysed bacteria (9). One of the most striking findings from our screen was the observation that, despite a wealth of data suggesting that DNA in the cytosol is sufficient to activate the AIM2 inflammasome (8, 9, 37, 38), a subset of our mutants which lyse and release both plasmid and chromosomal DNA into the cytosol of macrophages do not hyperactivate the inflammasome (Fig. 3). MK-deficient mutants lyse at frequencies similar to those seen with *pdhC*::*Tn*, *ΔyvcK* and *yvcJ*::*Tn* mutants but avoid activation of the AIM2 inflammasome. It is possible that during infection with MK mutants, changes in host metabolism activate host cell nucleases that modify or degrade bacterial DNA such that inflammasome signals are destroyed. Alternatively, it is possible that the response to infection with MK mutants leads to changes in signaling or cellular redox balance that inactivate either the AIM2 receptor or downstream signaling components, including caspase-1 itself (19, 39). Finally, although less likely given the ability of sterile transfected DNA to activate the AIM2 inflammasome, it is possible that DNA alone is not sufficient for AIM2 activation during infection and that non-respiring *L. monocytogenes* mutants lack the metabolic signals required to activate the inflammasome. Bacterial metabolism and regulation of innate immune signaling pathways appear to be intimately linked (19, 40–42). For example, S. Typhimurium metabolic mutants induce mitochondrial reactive oxygen production, resulting in NALP3-inflammasome activation (42). How MK-deficient mutants avoid inflammasome activation despite the release of cytosolic DNA is the subject of ongoing studies.

Previous studies performed with vacuolar pathogens that mislocalize to the cytosol highlighted differences between immune cells and nonimmune cells with respect to the ability to restrict pathogens in the cytosol. *S. enterica* Δ*sifA* mutants, which access the cytosol due to loss of vacuole integrity (43), appear to be killed in macrophages but not HeLa cells (3). *Legionella pneumophila* Δ*sdhA* mutants, which similarly mislocalize to the host cytosol (4), also display cell-specific survival defects (44) and are ultimately killed in the cytosol, resulting in activation of the AIM2 inflammasome (45). Our genetic screen also identified mutants with differential survival defects in different cell types and suggested that, similarly to the phagosomal killing capacity results, professional phagocytes such as macrophages may have more-potent cytosolic CADs (Fig. 1B). This may translate directly to virulence, as evidenced by the results seen with *pdhC*::*Tn* mutants, which were slowly cleared from macrophages but were capable of forming plaques in fibroblasts consistent with their improved survival in the cytosol of these cells (Fig. 2A and B). *L. monocytogenes* and other cytosolic pathogens likely employ various strategies to counter CADs in different cell types.

To kill cytosolic invaders, macrophages may utilize antimicrobial effectors such as ubiquicidin (46), interferon-inducible guanylate binding proteins (GBPs) (47), lysozyme (18), or autophagy (48). Unlike the results previously determined with *Francisella novicida* (47), recent data from our laboratory suggest that GBPs do not contribute to bacteriolysis of *L. monocytogenes* in the cytosol (49). Although the host factors which restrict cytosolic pathogens are unknown, a recent study found that *S. enterica* Δ*sifA* mutants experience oxidative and/or nitrosative stress in the macrophage cytosol (50), although it is unclear whether this stress originates from the host, from the bacterium, or from both. Our findings suggest that, despite increased sensitivity to ROS, cytosolic ROS is unlikely to be responsible for survival of *L. monocytogenes* MK-deficient mutants (Fig. S6C to E). Finally, in addition to identifying novel targets for therapeutic intervention, *L. monocytogenes* mutants susceptible to killing in the host cytosol act as tools to identify the host pathways responsible for killing these mutants.

Consistent with previous studies, *L. monocytogenes* MK-deficient mutants were highly attenuated for virulence *ex vivo* and *in vivo* (30, 31) (Fig. 2C and Fig. 4D and E); however, the function of MK itself, MK biosynthetic intermediates, or simply MK-dependent respiration in virulence has not been fully explored. Previous reports have found that MK biosynthesis genes and the cytochrome *bd* oxidase are upregulated during infection *ex vivo* (51) and *in vivo* (52), emphasizing the importance of MK for virulence. Here we uncovered a novel requirement for the MK intermediate DHNA in protecting *L. monocytogenes* from intracytosolic bacteriolysis (Fig. 6B and E). This phenotype was not due to disruption of the electron transport chain or loss of ATP generation through oxidative phosphorylation (Fig. 5D and F) but rather to the fact that DHNA synthesis is crucial for virulence and intracellular survival (Fig. 6B). Unfortunately, since the enzymatic steps at this stage in the pathway are incompletely understood (Fig. 6A) (35), we cannot determine whether DHNA or DHNA-CoA is the relevant molecule. Inexplicably, cytochrome *bd* oxidase and *menG*::*Tn* mutants displayed intermediate bacteriolysis phenotypes (Fig. 5D and F and Fig. 6B). Our favored hypothesis is that mutants with an incomplete ETC have depleted pools of available DHNA. This could be due either to increased funneling of DHNA into MK biosynthesis caused by incomplete electron transfer or, alternatively, to a transcriptional/posttranslation negative-feedback loop that inhibits DHNA synthesis in the absence of a productive ETC.

Apart from MK’s role in shuttling electrons between complexes in the electron transport chain, MK and its biosynthetic intermediates may play greater physiological roles in bacteria than previously appreciated. *Shewanella oneidensis* utilizes a derivative of DHNA to degrade carbon tetrachloride (53). In *S. aureus*, MK potentiates spermine and heme toxicity independently of MK’s function in the electron transport chain. Small-colony variants with mutations specifically in the MK biosynthesis pathway lead to increased resistance to spermine and heme (36, 54). Given that MK, DMK, and DHNA share naphthoquinone rings with similar redox characteristics, we surmise that these molecules may act as cofactors in chemical reactions crucial to the survival of *L. monocytogenes* in macrophages. It is also possible that *L. monocytogenes* uses DHNA as a substrate for synthesis of noncanonical MK (55) or another yet-to-be-characterized molecule. Alternatively, it has been proposed that two-component systems, such as ArcAB in *Escherichia coli* and SrrAB in *Staphylococcus aureus*, may sense changes in the redox state of MK as signals for environmental changes (56, 57). We posit that MK, DMK, and/or DHNA may be used as a sensor(s) of cytosolic stress. In the absence of these molecules, *L. monocytogenes* may not be able to adapt to this specific host environment.

Although decades of studies have focused on the host-pathogen interactions between macrophages and vacuolar pathogens, very little is known about how cytosolic pathogens survive in their primary niche and how the cell protects this environment from invasion by pathogens. Consistent with this study, previous work demonstrates that cytosolic survival of *Francisella* spp. relies on specific metabolic adaptations during invasion of the host cytosol (10), though the same metabolic pathways were not identified in this study. This outcome was expected since *L. monocytogenes* and *Francisella* spp. likely evolved mechanisms for cytosolic survival of the same host defense through convergent evolution. Here, we show that the MK biosynthetic intermediate DHNA promotes intracellular survival of *L. monocytogenes* in macrophages. These findings expand upon a series of recent studies demonstrating that central metabolism is at the nexus of host pathogen interactions. Host cells monitor and modulate their central metabolism both to sense and to defend against infection, whereas pathogens must modulate their metabolism both to make use of the available nutrients and to evade detection and survive in the face of host defenses. Finally, in addition to identification of a variety of new virulence determinants for *L. monocytogenes*, the mutants identified in this screen provide the tools necessary to identify host restriction factors that prevent cytosolic colonization by non-cytosol-adapted pathogens.

## MATERIALS AND METHODS

### Animals and cell cultures.

C57BL/6 mice were purchased from NCI. *Casp-1/-11*^*−/−*^ and *AIM2*^*−/−*^ mice were a kind gift from Russell Vance (University of California, Berkeley [UC Berkeley]) and Doug McNeel (University of Wisconsin—Madison [UW—Madison]), respectively. All experiments involving animals were performed according to protocols approved by the Animal Use and Care Committee of the University of Wisconsin—Madison.

*iIFNAR*^*−/−*^ macrophages and BHK and L2 cells were all kind gifts from Daniel Portnoy (UC Berkeley). Caco-2 cells were purchased from ATCC. Bone marrow-derived macrophages (BMDM) were prepared from 6-to-8-week-old mice as previously described (58).

### Bacterial strains, plasmid construction, and growth conditions *in vitro*.

*L. monocytogenes* 10403s was used as the wild-type strain. *L. monocytogenes* strains were grown at 37°C or 30°C in brain heart infusion (BHI), Luria broth (LB), or minimal defined media (min) supplemented with glucose as the sole carbon source (59). *Escherichia coli* strains were grown in Luria broth (LB) at 37°C. Antibiotics were used at a concentration of 100 µg/ml carbenicillin, 10 µg/ml chloramphenicol, 2 µg/ml erythromycin, or 30 µg/ml kanamycin when appropriate. For growth of MK-deficient strains in MK-limiting minimal media, MK (Sigma; V9378) was supplemented at 25 ng/ml. For anaerobic growth, bacteria were cultivated on BHI plates placed anaerobic jars carrying a GasPak EZ anaerobe container system (BD; 260678).

Vectors were shuttled into *L. monocytogenes* by the use of *E. coli* strain S17 or strain S10 through conjugation (60). In-frame deletions of genes in *L. monocytogenes* were performed by allelic exchange (61) using suicide plasmid pksv7-oriT as previously described (62). Integrative vector pIMK2 (63) was used for constitutive expression of *L. monocytogenes* genes. Transductions between *L. monocytogenes* strains were performed using U153 bacteriophage (64).

### Generation of transposon libraries and screening for bacteriolysis mutants.

The *Himar1* mariner transposon system was used to construct a random transposon library in wild-type *L. monocytogenes* carrying bacteriolysis reporter pBHE573 (8) as outlined in references 65 and 66. To perform the genetic screen, random mutant colonies from the *Himar1* bacteriolysis library were isolated and grown in wells from a 96-well plate overnight in BHI media at 30°C. In parallel, *iIFNAR*^*−/−*^ macrophages were seeded in opaque 96-well plates at 1 × 10^5^ cells per well and then allowed to incubate at 37°C in a 5% CO_2_ atmosphere for approximately 16 h. *iIFNAR*^*−/−*^ macrophages were infected at an estimated multiplicity of infection (MOI) of 10. At 1 h postinfection, the culture medium was exchanged for media containing 50 µg/ml gentamycin. At 6 h postinfection, the culture medium was replaced with TNT buffer to lyse cells. Luciferase reagent was added and measured for luciferase activity using a luminometer (BioTek Synergy HT).

### Intracellular bacteriolysis assay.

For standard bacteriolysis assays, unprimed *iIFNAR*^*−/−*^ macrophages, Caco-2 (epithelial) cells, or BHK (fibroblast) cells were plated at 5 × 10^5^ cells per well in a 24-well plate and then infected with *L. monocytogenes* strains carrying the plasmid bacteriolysis reporter (pBHE573) or chromosomal bacteriolysis reporter (pYL56) at an MOI of 10, 64, or 100, respectively. The higher MOIs used in the Caco-2 and BHK cells were used to gain comparable levels of luciferase production similar to those in macrophage experiments. At 1 h postinfection, cultures were treated with 50 µg/ml gentamicin. At 6 h postinfection (*iIFNAR*^*−/−*^ macrophages and BHK cells) or 8 h postinfection (Caco-2 cells), cells were assayed for luciferase activity as previously described (8). If indicated, infected cells were treated with 1 mg/ml ampicillin at 2 h postinfection.

### *In vitro* bacteriolysis assay.

Measurements of bacteriolysis *in vitro* were performed as previously described (28) with slight modifications. Cultures of *L. monocytogenes* carrying the *lacZ* gene encoding a transposon (Tn*917*-LTV3) were grown to mid-logarithmic or early stationary phase in BHI medium. Half of the culture was subjected to centrifugation to separate bacteria from the supernatant. The other half of the culture was subjected to complete bacteriolysis by addition of SDS to reach a concentration of 0.1% and to bead beating performed with 0.1-mm-diameter silicon beads for 10 min at 2,000 rpm. One hundred microliters of culture supernatant or 100 µl of serially diluted lysate (standard curve) was monitored for β-galactosidase activity by combining the supernatant or the lysate with 100 µl of 200 µM MUG (4-methylumbelliferyl β-d-galactopyranoside; Invitrogen) (excitation/emission [ex/em] wavelengths = 360 nm/449 nm) resuspended in Z buffer (0.1 M phosphate, 0.01 M KCl, 1 mM MgSO_4_, 50 mM β-mercaptoethanol, pH 7.0). The rate of β-galactosidase activity in the culture supernatant was compared to the standard curve to calculate percent bacteriolysis in the broth culture.

### Lactate dehydrogenase release.

BMDMs pretreated for 16 h with 100 ng/ml Pam3CSK4 (Invitrogen) were infected with *L. monocytogenes* at an MOI of 1. At 30 min postinfection, the medium was replaced with media containing 50 µg/ml gentamicin. At 6 h postinfection, supernatants were removed and measured for lactate dehydrogenase activity as previously described (67) using a BioTek Synergy HT spectrophotometer.

### Caspase-3/7 activity assay.

*iIFNAR*^*−/−*^ macrophages were seeded into white 96-well plates and infected with *L. monocytogenes* at an MOI of 10. Staurosporine (AG Scientific) (5 µM) was added to uninfected macrophages at 30 min postinfection to induce caspase-3/7 activation. At 6 h postinfection, caspase-3/7 activation was measured using a caspase-Glo 3/7 assay kit (Promega) according to the manufacturer’s instructions.

### Intracellular growth curves.

Wild-type or *IFNAR*^*−/−*^ BMDMs, Caco-2 cells, and BHK cells were infected with *L. monocytogenes* strains (at a multiplicity of infection [MOI] of 0.2 for the BMDMs and an MOI of 5 for the other cell types) and enumerated for CFU at various time points as previously described (58).

### L2 plaque assay.

Plaque assays were conducted using a L2 fibroblast cell line as previously described (32) with minor modifications for visualization and quantification of plaques. L2 fibroblasts were seeded at 1.2 × 10^6^ per 35-mm-diameter dish and infected at an MOI of 0.5 to obtain approximately 30 PFU per dish. At 4 to 6 days postinfection, cells were stained with 0.3% crystal violet for 10 min and washed twice with deionized water. Stained wells were imaged and areas of plaque formation were measured on Fiji image analysis software (68).

### Acute virulence assay.

Female C57BL/6 mice (6 to 8 weeks of age) were injected intravenously with 1 × 10^5^ CFU (50% lethal dose [LD_50_] = 1 for wild-type 10403s *L. monocytogenes*) of logarithmically growing *L. monocytogenes* (optical density at 600 nm [OD_600_] = 0.5). At 48 h postinfection, spleens and livers were harvested, homogenized, and enumerated for CFU as previously described (12).

### Measuring bacterial membrane potential.

*L. monocytogenes* strains were grown to mid-late-logarithmic phase in BHI media at 37°C. Bacteria were diluted in phosphate-buffered saline (PBS) to a concentration of 10^6^/ml and transferred to a fluorescence-activated cell sorter (FACS) tube. Next, cells were stained with 3 mM membrane potential indicator dye D_I_O_2_(3) (3,3′-diethyloxacarbocyanine iodide) (Sigma; 320684) and/or 500 µM proton ionophore CCCP (carbonyl cyanide 3-chlorophenylhydrazone) (Sigma; C2759) for 30 min. Samples were analyzed on a BD LSR-II flow cytometer, and data analysis was performed using FlowJo software. Samples were gated for bacteria using forward and side scatter, and then individual bacteria were measured for ratiometric fluorescence analysis using comparisons between red mean fluorescence intensity and green mean fluorescence intensity.

### MIC measurements.

*L. monocytogenes* strains were grown in aerated BHI cultures with various concentrations of ceftriaxone (Sigma; C5793), ampicillin (Sigma; A0166), bacitracin (Fisher Bioreagent; BP29501), daptomycin (Merck), lysozyme (Sigma; L6876), LL-37 (AnaSpec; AS61302), or hydrogen peroxide. MICs were determined as the median concentration of antimicrobial required to prevent replication over 12 h as determined by OD_600_ in a BioTek Eon or BioTek Synergy HT plate reader.

### Intracellular ROS measurements.

*iIFNAR*^*−/−*^ macrophages were grown in 35 mm-diameter dishes at a density of 2 × 10^6^ per dish. Cells were infected with *L. monocytogenes* at an MOI of 10 for 6 h or treated for 1 h with 100 µM menadione (positive control). N-acetylcysteine (NAC) (1 mM) was added to cell cultures throughout treatment to scavenge ROS. Treated cells were stained with 2.5 µM CellROX DeepRed (Molecular Probes; C10422) for 30 min at 37°C. Cells were then washed twice in FACS buffer, fixed with 3.7% formaldehyde for 15 min, and washed again prior to analysis on a BD LSR-II flow cytometer.

### Statistical analysis.

Statistical significance analysis (GraphPad Prism 6.0h) was performed using one-way or two-way analysis of variance (ANOVA) with the Bonferroni posttest unless otherwise indicated in the figure legends. Bacteriolysis assay data were log transformed prior to statistical analysis (*, P ≤ 0.05; **, P ≤ 0.01; ***, P* *≤ 0.001).

10.1128/mBio.00119-17.9 TABLE S2  Strains and plasmids used in this study. Download TABLE S2, DOCX file, 0.04 MB. Copyright © 2017 Chen et al. 2017 Chen et al. This content is distributed under the terms of the Creative Commons Attribution 4.0 International license.

10.1128/mBio.00119-17.10 TABLE S3  Primers used in this study. Download TABLE S3, DOCX file, 0.1 MB. Copyright © 2017 Chen et al. 2017 Chen et al. This content is distributed under the terms of the Creative Commons Attribution 4.0 International license.

## References

[1] Broz P, Monack DM 2013 Newly described pattern recognition receptors team up against intracellular pathogens. Nat Rev Immunol 13:551–565. doi:10.1038/nri3479. 23846113

[2] Goetz M, Bubert A, Wang G, Chico-Calero I, Vazquez-Boland JA, Beck M, Slaghuis J, Szalay AA, Goebel W 2001 Microinjection and growth of bacteria in the cytosol of mammalian host cells. Proc Natl Acad Sci U S A 98:12221–12226. doi:10.1073/pnas.211106398. 11572936 PMC59795

[3] Beuzón CR, Salcedo SP, Holden DW 2002 Growth and killing of a *Salmonella enterica* serovar Typhimurium *sifA* mutant strain in the cytosol of different host cell lines. Microbiology 148:2705–2715. doi:10.1099/00221287-148-9-2705. 12213917

[4] Creasey EA, Isberg RR 2012 The protein SdhA maintains the integrity of the *Legionella*-containing vacuole. Proc Natl Acad Sci U S A 109:3481–3486. doi:10.1073/pnas.1121286109. 22308473 PMC3295292

[5] Lecuit M 2007 Human listeriosis and animal models. Microbes Infect 9:1216–1225. doi:10.1016/j.micinf.2007.05.009. 17720601

[6] Freitag NE, Port GC, Miner MD 2009 *Listeria monocytogenes*—from saprophyte to intracellular pathogen. Nat Rev Microbiol 7:623–628. doi:10.1038/nrmicro2171. 19648949 PMC2813567

[7] Glomski IJ, Decatur AL, Portnoy DA 2003 *Listeria monocytogenes* mutants that fail to compartmentalize listeriolysin O activity are cytotoxic, avirulent, and unable to evade host extracellular defenses. Infect Immun 71:6754–6765. doi:10.1128/IAI.71.12.6754-6765.2003. 14638761 PMC308949

[8] Sauer JD, Witte CE, Zemansky J, Hanson B, Lauer P, Portnoy DA 2010 *Listeria monocytogenes* triggers AIM2-mediated pyroptosis upon infrequent bacteriolysis in the macrophage cytosol. Cell Host Microbe 7:412–419. doi:10.1016/j.chom.2010.04.004. 20417169 PMC2947455

[9] Jones JW, Kayagaki N, Broz P, Henry T, Newton K, O’Rourke K, Chan S, Dong J, Qu Y, Roose-Girma M, Dixit VM, Monack DM 2010 Absent in melanoma 2 is required for innate immune recognition of Francisella tularensis. Proc Natl Acad Sci U S A 107:9771–9776. doi:10.1073/pnas.1003738107. 20457908 PMC2906881

[10] Peng K, Broz P, Jones J, Joubert LM, Monack D 2011 Elevated AIM2‐mediated pyroptosis triggered by hypercytotoxic *Francisella* mutant strains is attributed to increased intracellular bacteriolysis. Cell Microbiol 13:1586–1600. doi:10.1111/j.1462-5822.2011.01643.x. 21883803 PMC3173570

[11] Tsuchiya K, Hara H, Kawamura I, Nomura T, Yamamoto T, Daim S, Dewamitta SR, Shen Y, Fang R, Mitsuyama M 2010 Involvement of absent in melanoma 2 in inflammasome activation in macrophages infected with *Listeria monocytogenes*. J Immunol 185:1186–1195. doi:10.4049/jimmunol.1001058. 20566831

[12] Sauer JD, Pereyre S, Archer KA, Burke TP, Hanson B, Lauer P, Portnoy DA 2011 *Listeria monocytogenes* engineered to activate the Nlrc4 inflammasome are severely attenuated and are poor inducers of protective immunity. Proc Natl Acad Sci U S A 108:12419–12424. doi:10.1073/pnas.1019041108. 21746921 PMC3145703

[13] Warren SE, Duong H, Mao DP, Armstrong A, Rajan J, Miao EA, Aderem A 2011 Generation of a *Listeria* vaccine strain by enhanced caspase-1 activation. Eur J Immunol 41:1934–1940. doi:10.1002/eji.201041214. 21538346 PMC3375905

[14] O’Riordan M, Moors MA, Portnoy DA 2003 Listeria intracellular growth and virulence require host-derived lipoic acid. Science 302:462–464. doi:10.1126/science.1088170. 14564012

[15] Joseph B, Przybilla K, Stühler C, Schauer K, Slaghuis J, Fuchs TM, Goebel W 2006 Identification of *Listeria monocytogenes* genes contributing to intracellular replication by expression profiling and mutant screening. J Bacteriol 188:556–568. doi:10.1128/JB.188.2.556-568.2006. 16385046 PMC1347271

[16] Chico-Calero I, Suárez M, González-Zorn B, Scortti M, Slaghuis J, Goebel W, Vázquez-Boland JA; European Listeria Genome Consortium 2002 Hpt, a bacterial homolog of the microsomal glucose-6-phosphate translocase, mediates rapid intracellular proliferation in listeria. Proc Natl Acad Sci U S A 99:431–436. doi:10.1073/pnas.012363899. 11756655 PMC117577

[17] Pensinger DA, Aliota MT, Schaenzer AJ, Boldon KM, Ansari IU, Vincent WJB, Knight B, Reniere ML, Striker R, Sauer JD 2014 Selective pharmacologic inhibition of a PASTA kinase increases Listeria monocytogenes susceptibility to β-lactam antibiotics. Antimicrob Agents Chemother 58:4486–4494. doi:10.1128/AAC.02396-14. 24867981 PMC4135996

[18] Rae CS, Geissler A, Adamson PC, Portnoy DA 2011 Mutations of the *Listeria monocytogenes* peptidoglycan N-deacetylase and O-acetylase result in enhanced lysozyme sensitivity, bacteriolysis, and hyperinduction of innate immune pathways. Infect Immun 79:3596–3606. doi:10.1128/IAI.00077-11. 21768286 PMC3165460

[19] Sanman LE, Qian Y, Eisele NA, Ng TM, van der Linden WA, Monack DM, Weerapana E, Bogyo M 2016 Disruption of glycolytic flux is a signal for inflammasome signaling and pyroptotic cell death. Elife 5:e13663. doi:10.7554/eLife.13663 27011353 PMC4846378

[20] Ivashkiv LB, Donlin LT 2014 Regulation of type I interferon responses. Nat Rev Immunol 14:36–49. doi:10.1038/nri3581. 24362405 PMC4084561

[21] Ofer A, Kreft J, Logan DT, Cohen G, Borovok I, Aharonowitz Y 2011 Implications of the inability of Listeria monocytogenes EGD-e to grow anaerobically due to a deletion in the class III NrdD ribonucleotide reductase for its use as a model laboratory strain. J Bacteriol 193:2931–2940. doi:10.1128/JB.01405-10. 21478338 PMC3133202

[22] Neveling U, Bringer-Meyer S, Sahm H 1998 Gene and subunit organization of bacterial pyruvate dehydrogenase complexes. Biochim Biophys Acta 1385:367–372. doi:10.1016/S0167-4838(98)00080-6. 9655937

[23] Meganathan R 2001 Biosynthesis of menaquinone (vitamin K2) and ubiquinone (coenzyme Q): a perspective on enzymatic mechanisms. Vitam Horm 61:173–218. doi:10.1016/S0083-6729(01)61006-9. 11153266

[24] Kazmierczak MJ, Mithoe SC, Boor KJ, Wiedmann M 2003 *Listeria monocytogenes* σ^B^ regulates stress response and virulence functions. J Bacteriol 185:5722–5734. doi:10.1128/JB.185.19.5722-5734.2003. 13129943 PMC193959

[25] Milohanic E, Glaser P, Coppée JY, Frangeul L, Vega Y, Vázquez-Boland JA, Kunst F, Cossart P, Buchrieser C 2003 Transcriptome analysis of *Listeria monocytogenes* identifies three groups of genes differently regulated by PrfA. Mol Microbiol 47:1613–1625. doi:10.1046/j.1365-2958.2003.03413.x. 12622816

[26] Wurtzel O, Sesto N, Mellin JR, Karunker I, Edelheit S, Bécavin C, Archambaud C, Cossart P, Sorek R 2012 Comparative transcriptomics of pathogenic and non-pathogenic *Listeria* species. Mol Syst Biol 8:583. doi:10.1038/msb.2012.11. 22617957 PMC3377988

[27] Camilli A, Paynton CR, Portnoy DA 1989 Intracellular methicillin selection of *Listeria monocytogenes* mutants unable to replicate in a macrophage cell line. Proc Natl Acad Sci U S A 86:5522–5526. doi:10.1073/pnas.86.14.5522. 2501788 PMC297655

[28] Witte CE, Whiteley AT, Burke TP, Sauer JD, Portnoy DA, Woodward JJ 2013 Cyclic di-AMP is critical for *Listeria monocytogenes* growth, cell wall homeostasis, and establishment of infection. mBio 4:e00282-13. doi:10.1128/mBio.00282-13. 23716572 PMC3663569

[29] Whiteley AT, Pollock AJ, Portnoy DA 2015 The PAMP c-di-AMP Is essential for *Listeria monocytogenes* growth in rich but not minimal media due to a toxic increase in (p)ppGpp. Cell Host Microbe 17:788–798. doi:10.1016/j.chom.2015.05.006. 26028365 PMC4469362

[30] Stritzker J, Janda J, Schoen C, Taupp M, Pilgrim S, Gentschev I, Schreier P, Geginat G, Goebel W 2004 Growth, virulence, and immunogenicity of *Listeria monocytogenes* aro mutants. Infect Immun 72:5622–5629. doi:10.1128/IAI.72.10.5622-5629.2004. 15385459 PMC517589

[31] Perry KJ, Higgins DE 2013 A differential fluorescence-based genetic screen identifies *Listeria monocytogenes* determinants required for intracellular replication. J Bacteriol 195:3331–3340. doi:10.1128/JB.00210-13. 23687268 PMC3719552

[32] Sun AN, Camilli A, Portnoy DA 1990 Isolation of Listeria monocytogenes small-plaque mutants defective for intracellular growth and cell-to-cell spread. Infect Immun 58:3770–3778. 2172168 10.1128/iai.58.11.3770-3778.1990 PMC313727

[33] Glaser P, Frangeul L, Buchrieser C, Rusniok C, Amend A, Baquero F, Berche P, Bloecker H, Brandt P, Chakraborty T, Charbit A, Chetouani F, Couvé E, de Daruvar A, Dehoux P, Domann E, Domínguez-Bernal G, Duchaud E, Durant L, Dussurget O, Entian KD, Fsihi H, García-del Portillo F, Garrido P, Gautier L, Goebel W, Gómez-López N, Hain T, Hauf J, Jackson D, Jones LM, Kaerst U, Kreft J, Kuhn M, Kunst F, Kurapkat G, Madueno E, Maitournam A, Vicente JM, Ng E, Nedjari H, Nordsiek G, Novella S, de Pablos B, Pérez-Diaz JC, Purcell R, Remmel B, Rose M, Schlueter T, 2001 Comparative genomics of *Listeria* species. Science 294:849–852. doi:10.1126/science.1063447. 11679669

[34] Yoshida M, Muneyuki E, Hisabori T 2001 ATP synthase—a marvellous rotary engine of the cell. Nat Rev Mol Cell Biol 2:669–677. doi:10.1038/35089509. 11533724

[35] Chen M, Ma X, Chen X, Jiang M, Song H, Guo Z 2013 Identification of a hotdog fold thioesterase involved in the biosynthesis of menaquinone in *Escherichia coli*. J Bacteriol 195:2768–2775. doi:10.1128/JB.00141-13. 23564174 PMC3697248

[36] Wakeman CA, Hammer ND, Stauff DL, Attia AS, Anzaldi LL, Dikalov SI, Calcutt MW, Skaar EP 2012 Menaquinone biosynthesis potentiates haem toxicity in *Staphylococcus aureus*. Mol Microbiol 86:1376–1392. doi:10.1111/mmi.12063. 23043465 PMC3524387

[37] Hornung V, Ablasser A, Charrel-Dennis M, Bauernfeind F, Horvath G, Caffrey DR, Latz E, Fitzgerald KA 2009 AIM2 recognizes cytosolic dsDNA and forms a caspase-1-activating inflammasome with ASC. Nature 458:514–518. doi:10.1038/nature07725. 19158675 PMC2726264

[38] Fernandes-Alnemri T, Yu JW, Datta P, Wu J, Alnemri ES 2009 AIM2 activates the inflammasome and cell death in response to cytoplasmic DNA. Nature 458:509–513. doi:10.1038/nature07710. 19158676 PMC2862225

[39] Mishra BB, Rathinam VA, Martens GW, Martinot AJ, Kornfeld H, Fitzgerald KA, Sassetti CM 2013 Nitric oxide controls the immunopathology of tuberculosis by inhibiting NLRP3 inflammasome-dependent processing of IL-1β Nat Immunol 14:52–60. doi:10.1038/ni.2474. 23160153 PMC3721324

[40] Gaudet RG, Sintsova A, Buckwalter CM, Leung N, Cochrane A, Li J, Cox AD, Moffat J, Gray-Owen SD 2015 Cytosolic detection of the bacterial metabolite HBP activates TIFA-dependent innate immunity. Science 348:1251–1255. doi:10.1126/science.aaa4921. 26068852

[41] Woodward JJ, Iavarone AT, Portnoy DA 2010 c-di-AMP secreted by intracellular *Listeria monocytogenes* activates a host type I interferon response. Science 328:1703–1705. doi:10.1126/science.1189801. 20508090 PMC3156580

[42] Wynosky-Dolfi MA, Snyder AG, Philip NH, Doonan PJ, Poffenberger MC, Avizonis D, Zwack EE, Riblett AM, Hu B, Strowig T, Flavell RA, Jones RG, Freedman BD, Brodsky IE 2014 Oxidative metabolism enables *Salmonella* evasion of the NLRP3 inflammasome. J Exp Med 211:653–668. doi:10.1084/jem.20130627. 24638169 PMC3978275

[43] Beuzón CR, Méresse S, Unsworth KE, Ruíz-Albert J, Garvis S, Waterman SR, Ryder TA, Boucrot E, Holden DW 2000 *Salmonella* maintains the integrity of its intracellular vacuole through the action of SifA. EMBO J 19:3235–3249. doi:10.1093/emboj/19.13.3235. 10880437 PMC313946

[44] Laguna RK, Creasey EA, Li Z, Valtz N, Isberg RR 2006 A Legionella pneumophila-translocated substrate that is required for growth within macrophages and protection from host cell death. Proc Natl Acad Sci U S A 103:18745–18750. doi:10.1073/pnas.0609012103. 17124169 PMC1656969

[45] Ge J, Gong YN, Xu Y, Shao F 2012 Preventing bacterial DNA release and absent in melanoma 2 inflammasome activation by a *Legionella* effector functioning in membrane trafficking. Proc Natl Acad Sci U S A 109:6193–6198. doi:10.1073/pnas.1117490109. 22474394 PMC3341053

[46] Hiemstra PS, van den Barselaar MT, Roest M, Nibbering PH, van Furth R 1999 Ubiquicidin, a novel murine microbicidal protein present in the cytosolic fraction of macrophages. J Leukoc Biol 66:423–428. 10496312 10.1002/jlb.66.3.423

[47] Meunier E, Wallet P, Dreier RF, Costanzo S, Anton L, Rühl S, Dussurgey S, Dick MS, Kistner A, Rigard M, Degrandi D, Pfeffer K, Yamamoto M, Henry T, Broz P 2015 Guanylate-binding proteins promote activation of the AIM2 inflammasome during infection with *Francisella novicida*. Nat Immunol 16:476–484. doi:10.1038/ni.3119. 25774716 PMC4568307

[48] Knodler LA, Celli J 2011 Eating the strangers within: host control of intracellular bacteria via xenophagy. Cell Microbiol 13:1319–1327. doi:10.1111/j.1462-5822.2011.01632.x. 21740500 PMC3158265

[49] Pensinger DA, Boldon KM, Chen GY, Vincent WJB, Sherman K, Xiong M, Schaenzer AJ, Forster ER, Coers J, Striker R, Sauer JD 2016 The *Listeria monocytogenes* PASTA kinase PrkA and its substrate YvcK are required for cell wall homeostasis, metabolism, and virulence. PLoS Pathog 12:e1006001. doi:10.1371/journal.ppat.1006001. 27806131 PMC5091766

[50] van der Heijden J, Bosman ES, Reynolds LA, Finlay BB 2015 Direct measurement of oxidative and nitrosative stress dynamics in *Salmonella* inside macrophages. Proc Natl Acad Sci U S A 112:560–565. doi:10.1073/pnas.1414569112. 25548165 PMC4299202

[51] Chatterjee SS, Hossain H, Otten S, Kuenne C, Kuchmina K, Machata S, Domann E, Chakraborty T, Hain T 2006 Intracellular gene expression profile of *Listeria monocytogenes*. Infect Immun 74:1323–1338. doi:10.1128/IAI.74.2.1323-1338.2006. 16428782 PMC1360297

[52] Camejo A, Buchrieser C, Couvé E, Carvalho F, Reis O, Ferreira P, Sousa S, Cossart P, Cabanes D 2009 In vivo transcriptional profiling of *Listeria monocytogenes* and mutagenesis identify new virulence factors involved in infection. PLoS Pathog 5:e1000449. doi:10.1371/journal.ppat.1000449. 19478867 PMC2679221

[53] Ward MJ, Fu QS, Rhoads KR, Yeung CHJ, Spormann AM, Criddle CS 2004 A derivative of the menaquinone precursor 1,4-dihydroxy-2-naphthoate is involved in the reductive transformation of carbon tetrachloride by aerobically grown *Shewanella oneidensis* MR-1. Appl Microbiol Biotechnol 63:571–577. doi:10.1007/s00253-003-1407-3. 12908086

[54] Joshi GS, Spontak JS, Klapper DG, Richardson AR 2011 Arginine catabolic mobile element encoded *speG* abrogates the unique hypersensitivity of Staphylococcus aureus to exogenous polyamines. Mol Microbiol 82:9–20. doi:10.1111/j.1365-2958.2011.07809.x. 21902734 PMC3183340

[55] Holsclaw CM, Sogi KM, Gilmore SA, Schelle MW, Leavell MD, Bertozzi CR, Leary JA 2008 Structural characterization of a novel sulfated menaquinone produced by *stf3* from *Mycobacterium tuberculosis*. ACS Chem Biol 3:619–624. doi:10.1021/cb800145r. 18928249 PMC2680727

[56] Bekker M, Alexeeva S, Laan W, Sawers G, Teixeira de Mattos JT, Hellingwerf K 2010 The ArcBA two-component system of *Escherichia coli* is regulated by the redox state of both the ubiquinone and the menaquinone pool. J Bacteriol 192:746–754. doi:10.1128/JB.01156-09. 19933363 PMC2812447

[57] Kinkel TL, Roux CM, Dunman PM, Fang FC 2013 The *Staphylococcus aureus* SrrAB two-component system promotes resistance to nitrosative stress and hypoxia. mBio 4:e00696doi: 10.1128/mBio.00696-13. 24222487 PMC3892780

[58] Jones S, Portnoy DA 1994 Characterization of *Listeria monocytogenes* pathogenesis in a strain expressing perfringolysin O in place of listeriolysin O. Infect Immun 62:5608–5613. 7960143 10.1128/iai.62.12.5608-5613.1994 PMC303309

[59] Phan-Thanh L, Gormon T 1997 A chemically defined minimal medium for the optimal culture of *Listeria*. Int J Food Microbiol 35:91–95. doi:10.1016/S0168-1605(96)01205-6. 9081230

[60] Simon R, Priefer U, Pühler A 1983 A broad host range mobilization system for in vivo genetic engineering: transposon mutagenesis in Gram negative bacteria. Nat Biotechnol 1:784–791. doi:10.1038/nbt1183-784.

[61] Camilli A, Tilney LG, Portnoy DA 1993 Dual roles of *plcA* in *Listeria monocytogenes* pathogenesis. Mol Microbiol 8:143–157. doi:10.1111/j.1365-2958.1993.tb01211.x. 8388529 PMC4836944

[62] Lauer P, Hanson B, Lemmens EE, Liu W, Luckett WS, Leong ML, Allen HE, Skoble J, Bahjat KS, Freitag NE, Brockstedt DG, Dubensky TW 2008 Constitutive activation of the PrfA regulon enhances the potency of vaccines based on live-attenuated and killed but metabolically active *Listeria monocytogenes* strains. Infect Immun 76:3742–3753. doi:10.1128/IAI.00390-08. 18541651 PMC2493200

[63] Monk IR, Gahan CGM, Hill C 2008 Tools for functional postgenomic analysis of *Listeria monocytogenes*. Appl Environ Microbiol 74:3921–3934. doi:10.1128/AEM.00314-08. 18441118 PMC2446514

[64] Hodgson DA 2000 Generalized transduction of serotype 1/2 and serotype 4b strains of *Listeria monocytogenes*. Mol Microbiol 35:312–323. doi:10.1046/j.1365-2958.2000.01643.x. 10652092

[65] Cao M, Bitar AP, Marquis H 2007 A mariner-based transposition system for *Listeria monocytogenes*. Appl Environ Microbiol 73:2758–2761. doi:10.1128/AEM.02844-06. 17308180 PMC1855599

[66] Zemansky J, Kline BC, Woodward JJ, Leber JH, Marquis H, Portnoy DA 2009 Development of a mariner-based transposon and identification of *Listeria monocytogenes* determinants, including the peptidyl-prolyl isomerase PrsA2, that contribute to its hemolytic phenotype. J Bacteriol 191:3950–3964. doi:10.1128/JB.00016-09. 19376879 PMC2698408

[67] Decker T, Lohmann-Matthes ML 1988 A quick and simple method for the quantitation of lactate dehydrogenase release in measurements of cellular cytotoxicity and tumor necrosis factor (TNF) activity. J Immunol Methods 115:61–69. doi:10.1016/0022-1759(88)90310-9. 3192948

[68] Schindelin J, Arganda-Carreras I, Frise E, Kaynig V, Longair M, Pietzsch T, Preibisch S, Rueden C, Saalfeld S, Schmid B, Tinevez JY, White DJ, Hartenstein V, Eliceiri K, Tomancak P, Cardona A 2012 Fiji: an open-source platform for biological-image analysis. Nat Methods 9:676–682. doi:10.1038/nmeth.2019. 22743772 PMC3855844

