# Supplementary material for: A Genetic Screen Reveals that Synthesis of 1,4-Dihydroxy-2-Naphthoate (DHNA), but Not Full-Length Menaquinone, Is Required for Listeria monocytogenes Cytosolic Survival
Source: mBio. 2017 Mar 21;8(2):e00119-17. doi: 10.1128/mBio.00119-17 (PMC5362031; doi:10.1128/mBio.00119-17)
Supplement: TABLE S1 [file mbo002173238st1.docx]

| **lmo #** | **Gene** | **Insertion** | **Function** |
| --- | --- | --- | --- |
| *0279* | *nrdD* | +259 | anaerobic ribonucleoside triphosphate reductase |
| *1054* | *pdhC* | +419 | highly similar to pyruvate dehydrogenase (dihydrolipoamide acetyltransferase E2 subunit) |
| *1602* | - | +64 | hypothetical protein |
| *1675* | *menD* | +227 | similar to 2-succinyl-6-hydroxy-2,4-cyclohexadiene-1-carboxylate synthase / 2-oxoglutarate decarboxylase |
| *1676* | *menF* | +937 | similar to menaquinone-specific isochorismate synthase |
| *2474* | *yvcJ* | +17 | hypothetical protein |
