# Supplementary figures and images for: A Genetic Screen Reveals that Synthesis of 1,4-Dihydroxy-2-Naphthoate (DHNA), but Not Full-Length Menaquinone, Is Required for Listeria monocytogenes Cytosolic Survival
Source: mBio. 2017 Mar 21;8(2):e00119-17. doi: 10.1128/mBio.00119-17 (PMC5362031; doi:10.1128/mBio.00119-17)

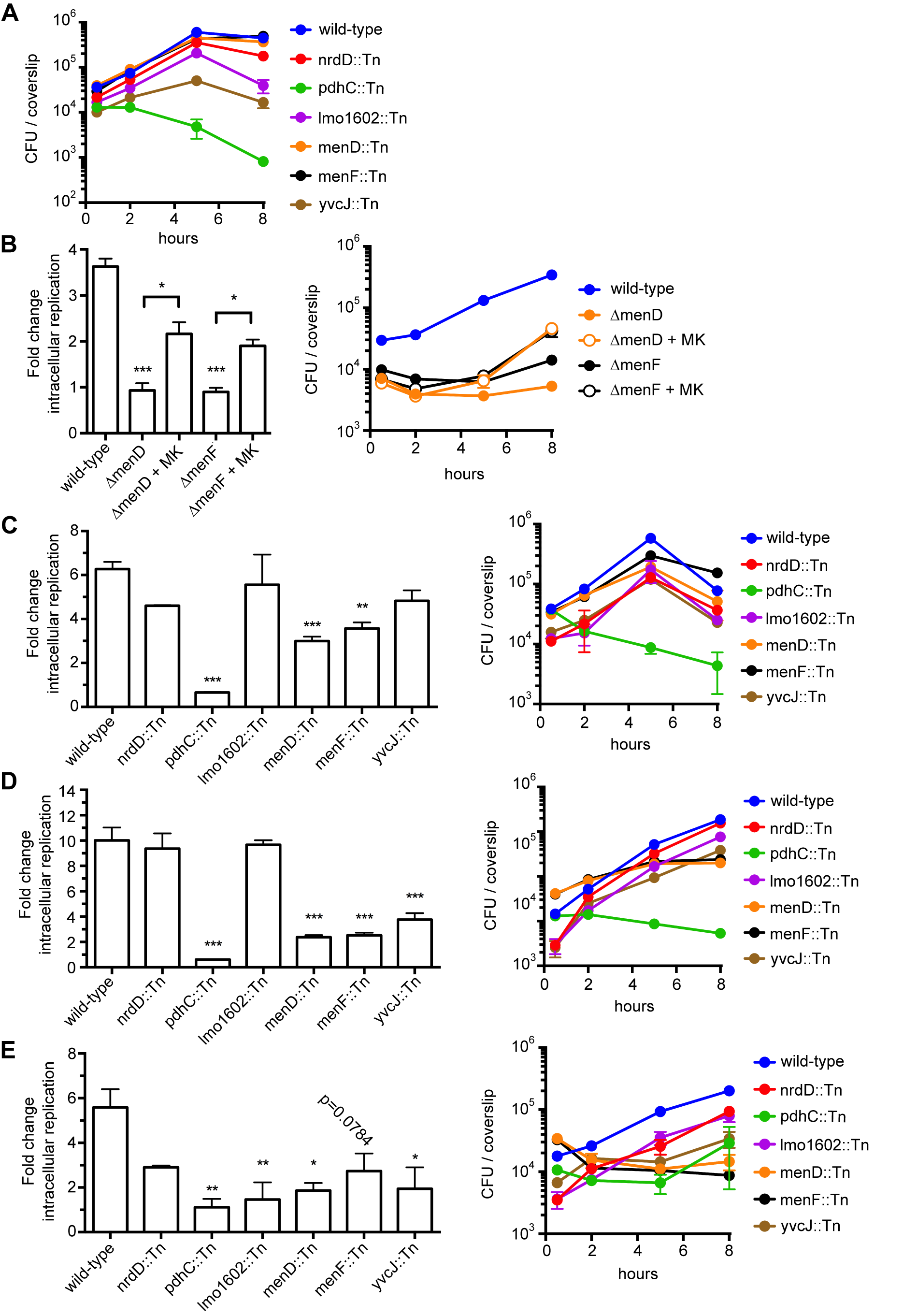

Supplement: FIG S4 [file mbo002173238sf4.tif]
