# Supplementary material for: A Genetic Screen Reveals that Synthesis of 1,4-Dihydroxy-2-Naphthoate (DHNA), but Not Full-Length Menaquinone, Is Required for Listeria monocytogenes Cytosolic Survival
Source: mBio. 2017 Mar 21;8(2):e00119-17. doi: 10.1128/mBio.00119-17 (PMC5362031; doi:10.1128/mBio.00119-17)
Supplement: TABLE S2 [file mbo002173238st2.docx]

| **Strain** | **Description** | **Reference** |
| --- | --- | --- |
| XL1-Blue | competent *E. coli* strain | - |
| SM10 | *E. coli* strain for conjugations into *Lm;* Km^R^ | (1) |
| S17 | *E. coli* strain for conjugations into *Lm;* Sp^R^ | (1) |
| 10403S | parental *Lm* 10403s strain | - |
| DP-L5932 | *Lm c∆dacA* [*dacA* conditional mutant] | (2) |
| JDS816 | *Lm c∆dacA* Tn917-LTV3(*lacZ*) | this work |
| DP-L967 | *Lm* Tn917-LTV3(*lacZ)* | (3) |
| JDS18 | *Lm* with pBHE573 | (4) |
| JDS20 | *Lm* expressing holin/lysin and with pBHE573 | (4) |
| JDS893 | *Lm* pYL56 | this work |
| JDS960 | *Lm* ∆*cydAB* | this work |
| JDS955 | *Lm* ∆*cydAB* with pBHE573 | this work |
| JDS1119 | *Lm* ∆*cydAB/*∆*qoxA* | this work |
| JDS1185 | *Lm* ∆*cydAB/*∆*qoxA* with pBHE573 | this work |
| JDS895 | *Lm* ∆*hly* pYL56 | this work |
| JDS19 | *Lm* ∆hly with pBHE573 | (4) |
| JDS1218 | *Lm* ∆*lmo1602* | this work |
| JDS1131 | *Lm* ∆*lmo1602* with pIMK2 | this work |
| JDS1198 | *Lm* ∆*lmo1602* with pIMK2 and pBHE573 | this work |
| JDS1132 | *Lm* ∆*lmo1602* with pYL112 | this work |
| JDS1199 | *Lm* ∆*lmo1602* with pYL112 and pBHE573 | this work |
| JDS1047 | *Lm* ∆*menA* | this work |
| JDS1073 | *Lm* ∆*menA* with pBHE573 | this work |
| JDS1164 | *Lm* ∆*menA/*∆*menB* | this work |
| JDS1212 | *Lm* ∆*menA/*∆*menB* with pBHE573 | this work |
| JDS1161 | *Lm* ∆*menB* | this work |
| JDS1191 | *Lm* ∆*menB* with pBHE573 | this work |
| JDS1213 | *Lm* ∆*menD* | (5) |
| JDS1213 | *Lm* ∆*menD* | this work |
| JDS896 | *Lm* ∆*menD* pYL56 | this work |
| JDS431 | *Lm* ∆*menD* with pBHE573 | this work |
| JDS667 | *Lm* ∆*menD* with pIMK2 | this work |
| JDS667 | *Lm ∆menD* with pIMK2 | this work |
| JDS669 | *Lm ∆menD* with pIMK2 and pBHE573 | this work |
| JDS669 | *Lm* ∆*menD* with pIMK2 and pBHE573 | this work |
| JDS611 | *Lm* ∆*menD* with pYL48 | this work |
| JDS666 | *Lm ∆menD* with pYL48 and pBHE573 | this work |
| JDS666 | *Lm* ∆*menD* with pYL48 and pBHE573 | this work |
| JDS608 | *Lm* ∆*menF* | this work |
| JDS608 | *Lm* ∆*menF* | this work |
| JDS665 | *Lm ∆menF* complemented with *menF* and pBHE573 | this work |
| JDS817 | *Lm ∆menF* Tn917-LTV3(*lacZ*) | this work |
| JDS618 | *Lm* ∆*menF* with pBHE573 | this work |
| JDS668 | *Lm* ∆*menF* with pIMK2 | this work |
| JDS668 | *Lm ∆menF* with pIMK2 | this work |
| JDS670 | *Lm ∆menF* with pIMK2 and pBHE573 | this work |
| JDS670 | *Lm* ∆*menF* with pIMK2 and pBHE573 | this work |
| JDS612 | *Lm* ∆*menF* with pYL44 | this work |
| JDS665 | *Lm* ∆*menF* with pYL44 and pBHE573 | this work |
| JDS1217 | *Lm* ∆*nrdD* | this work |
| JDS1058 | *Lm* ∆*nrdD* with pIMK2 | this work |
| JDS1084 | *Lm* ∆*nrdD* with pIMK2 and pBHE573 | this work |
| JDS1050 | *Lm* ∆*nrdD* with pYL107 | this work |
| JDS1075 | *Lm* ∆*nrdD* with pYL107 and pBHE573 | this work |
| JDS1049 | *Lm* ∆*qoxA* | this work |
| JDS1184 | *Lm* ∆*qoxA* with pBHE573 | this work |
| JDS1219 | *Lm* ∆*yvcJ* | this work |
| JDS1060 | *Lm* ∆*yvcJ* with pIMK2 | this work |
| JDS1086 | *Lm* ∆*yvcJ* with pIMK2 and pBHE573 | this work |
| JDS894 | *Lm* ∆*yvcK* pYL56 | this work |
| JDS21 | *Lm ∆yvcK* with pBHE573 | (4) |
| JDS1209 | *Lm 1602::Tn* | this work |
| JDS1254 | *Lm 1602::Tn* Tn917-LTV3(*lacZ*) | this work |
| DP-L5702 | *Lm atpH::Tn* | (6) |
| JDS755 | *Lm atpH::Tn* with pBHE573 | this work |
| JDS723 | *Lm cydA::Tn* | this work |
| JDS735 | *Lm cydA::Tn* with pBHE573 | this work |
| JDS903 | *Lm lmo1602::Tn* pYL56 | this work |
| JDS1266 | *Lm lmo1602::Tn* with pBHE573 | this work |
| JDS1198 | *Lm lmo1602::Tn* with pIMK2 and pBHE573 | this work |
| JDS1129 | *Lm lmo1602::Tn* with pYL112 | this work |
| JDS1200 | *Lm lmo1602::Tn* with pYL112 and pBHE573 | this work |
| JDS725 | *Lm menB::Tn* | this work |
| JDS737 | *Lm menB::Tn* with pBHE573 | this work |
| JDS726 | *Lm menC::Tn* | this work |
| JDS738 | *Lm menC::Tn* with pBHE573 | this work |
| JDS1204 | *Lm menD::Tn* | this work |
| JDS898 | *Lm menD::Tn* pYL56 | this work |
| JDS1249 | *Lm menD::Tn* Tn917-LTV3(*lacZ*) | this work |
| JDS1261 | *Lm menD::Tn* with pBHE573 | this work |
| JDS1194 | *Lm menD::Tn* with pIMK2 and pBHE573 | this work |
| JDS1181 | *Lm menD::Tn* with pYL48 | this work |
| JDS1195 | *Lm menD::Tn* with pYL48 and pBHE573 | this work |
| JDS727 | *Lm menE::Tn* | this work |
| JDS739 | *Lm menE::Tn* with pBHE573 | this work |
| JDS1206 | *Lm menF::Tn* | this work |
| JDS900 | *Lm menF::Tn* pYL56 | this work |
| JDS1251 | *Lm menF::Tn* Tn917-LTV3(*lacZ*) | this work |
| JDS126 | *Lm menF::Tn* with pBHE573 | this work |
| JDS1196 | *Lm menF::Tn* with pIMK2 and pBHE573 | this work |
| JDS1169 | *Lm menF::Tn* with pYL44 | this work |
| JDS1197 | *Lm menF::Tn* with pYL44 and pBHE573 | this work |
| JDS728 | *Lm menG::Tn* | this work |
| JDS740 | *Lm menG::Tn* with pBHE573 | this work |
| JDS1208 | *Lm nrdD::Tn* | this work |
| JDS902 | *Lm nrdD::Tn* pYL56 | this work |
| JDS1253 | *Lm nrdD::Tn* Tn917-LTV3(*lacZ*) | this work |
| JDS1265 | *Lm nrdD::Tn* with pBHE573 | this work |
| JDS1188 | *Lm nrdD::Tn* with pIMK2 and pBHE573 | this work |
| JDS1173 | *Lm nrdD::Tn* with pYL107 | this work |
| JDS1187 | *Lm nrdD::Tn* with pYL107 and pBHE573 | this work |
| JDS1203 | *Lm pdhC::Tn* | this work |
| JDS1064 | *Lm pdhC::Tn* pYL106 | this work |
| JDS1081 | *Lm pdhC::Tn* pYL106 and pBHE573 | this work |
| JDS897 | *Lm pdhC::Tn* pYL56 | this work |
| JDS1248 | *Lm pdhC::Tn* Tn917-LTV3(*lacZ*) | this work |
| JDS1260 | *Lm pdhC::Tn* with pBHE573 | this work |
| JDS1088 | *Lm pdhC::Tn* with pIMK2 and pBHE573 | this work |
| DP-L5640 | *Lm qoxA::Tn* | (6) |
| JDS754 | *Lm qoxA::Tn* with pBHE573 | this work |
| JDS1210 | *Lm yvcJ::Tn* | this work |
| JDS904 | *Lm yvcJ::Tn* pYL56 | this work |
| JDS1255 | *Lm yvcJ::Tn* Tn917-LTV3(*lacZ*) | this work |
| JDS1267 | *Lm yvcJ::Tn* with pBHE573 | this work |

| **Plasmids** | **Description** | **Reference** |
| --- | --- | --- |
| pBHE573 | bacteriolysis reporter; Cm^R^ | (4) |
| pIMK2 | expression vector, P_help_; Km^R^ | (7) |
| pJZ037 | for construction of *Himar1* library; Ap^R^ | (8) |
| pKSV7-oriT | for generation of clean deletions; Ap^R^ | (9) |
| pYL10 | *Lm ∆*nrdD construct ligated into pKSV7-oriT | this work |
| pYL106 | *Lm pdhC* ligated into pIMK2 | this work |
| pYL107 | *Lm nrdD* ligated into pIMK2 | this work |
| pYL112 | *Lm lmo1602* ligated into pIMK2 | this work |
| pYL115 | *Lm ∆menB* construct ligated into pKSV7-oriT | this work |
| pYL117 | *Lm ∆menA* construct ligated into pKSV7-oriT | this work |
| pYL34 | *Lm ∆menF* construct ligated into pksv7-oriT | this work |
| pYL44 | *Lm menF* ligated into pIMK2 | this work |
| pYL48 | *Lm menD* ligated into pIMK2 | this work |
| pYL56 | chromosomal bacteriolysis reporter | this work |
| pYL67 | *Lm ∆cydAB* construct ligated into pKSV7-oriT | this work |
| pYL97 | *Lm ∆qoxA* construct ligated into pKSV7-oriT | this work |
| pYL98 | *Lm ∆lmo1602* construct ligated into pKSV7-oriT | this work |

**SI REFENCES**

1. **Simon R**, **Priefer U**, **Pühler A**. 1983. A broad host range mobilization system for in vivo genetic engineering: transposon mutagenesis in Gram negative bacteria. Nat Biotechnol **1**:784–791.

2. **Witte CE**, **Whiteley AT**, **Burke TP**. 2013. Cyclic di-AMP Is critical for *Listeria monocytogenes* growth, cell wall homeostasis, and establishment of infection. MBio **4**:1–10.

3. **Sun AN**, **Camilli A**, **Portnoy DA**. 1990. Isolation of Listeria monocytogenes small-plaque mutants defective for intracellular growth and cell-to-cell spread. Infect Immun **58**:3770–3778.

4. **Sauer J-D**, **Witte CE**, **Zemansky J**, **Hanson B**, **Lauer P**, **Portnoy DA**. 2010. *Listeria monocytogenes* triggers AIM2-mediated pyroptosis upon infrequent bacteriolysis in the macrophage cytosol. Cell Host Microbe **7**:412–419.

5. **Perry KJ**, **Higgins DE**. 2013. A differential fluorescence-based genetic screen identifies *Listeria monocytogenes* determinants required for intracellular replication. J Bacteriol **195**:3331–3340.

6. **McKay SL**, **Portnoy DA**. 2015. Ribosome hibernation facilitates tolerance of stationary-phase bacteria to aminoglycosides. Antimicrob Agents Chemother **59**:6992–6999.

7. **Monk IR**, **Gahan CGM**, **Hill C**. 2008. Tools for functional postgenomic analysis of *Listeria monocytogenes*. Appl Environ Microbiol **74**:3921–3934.

8. **Zemansky J**, **Kline BC**, **Woodward JJ**, **Leber JH**, **Marquis H**, **Portnoy DA**. 2009. Development of a mariner-based transposon and identification of *Listeria monocytogenes* determinants, including the peptidyl-prolyl isomerase PrsA2, that contribute to its hemolytic phenotype. J Bacteriol2009/04/21. **191**:3950–3964.

9. **Lauer P**, **Hanson B**, **Lemmens EE**, **Liu W**, **Luckett WS**, **Leong ML**, **Allen HE**, **Skoble J**, **Bahjat KS**, **Freitag NE**, **Brockstedt DG**, **Dubensky TW**. 2008. Constitutive activation of the PrfA regulon enhances the potency of vaccines based on live-attenuated and killed but metabolically active *Listeria monocytogenes* strains. Infect Immun **76**:3742–3753.
