# Supplementary material for: A Genetic Screen Reveals that Synthesis of 1,4-Dihydroxy-2-Naphthoate (DHNA), but Not Full-Length Menaquinone, Is Required for Listeria monocytogenes Cytosolic Survival
Source: mBio. 2017 Mar 21;8(2):e00119-17. doi: 10.1128/mBio.00119-17 (PMC5362031; doi:10.1128/mBio.00119-17)
Supplement: TABLE S3 [file mbo002173238st3.docx]

| **Primers** | **Sequence** | **Description** | **Restriction Sites** |
| --- | --- | --- | --- |
| GC56B | AGGAGAGATAAGCATATGAATACTAAATTGGGCATAAAGAAATGACAAACCACGAACA | construct *menF* deletion; SOE primer B |  |
| GC56C | TGTTCGTGGTTTGTCATTTCTTTATGCCCAATTTAGTATTCATATGCTTATCTCTCCT | construct *menF* deletion; SOE primer C |  |
| GC57B | CACACGGTTTTGGATTATTCACCTAAGTTTTGTTCCACGTACATGATAATCCCCC | construct *nrdD* deletion; SOE primer B |  |
| GC57C | GGGGGATTATCATGTACGTGGAACAAAACTTAGGTGAATAATCCAAAACCGTGTG | construct *nrdD* deletion; SOE primer C |  |
| GC59B | AGGTGTGGATAGATGATAGTAATCTTGCAATCTAATTTCTAATATAATTTGGAGGGATTT | construct *lmo1602* deletion; SOE primer B |  |
| GC59C | AAATCCCTCCAAATTATATTAGAAATTAGATTGCAAGATTACTATCATCTATCCACACCT | construct *lmo1602* deletion; SOE primer C |  |
| GC104A | ATATTAGGTACCGATACGAGTTGCCATTGTC | construct *nrdD* deletion; SOE primer A | KpnI |
| GC104D | ATATTAGAGCTCCATTTGCACCCATCACTAAAG | construct *nrdD* deletion; SOE primer D | SacI |
| GC157A | ATATTAGGTACCAAAATCGGTACAAGCGGTTC | construct *menF* deletion; SOE primer A | KpnI |
| GC157D | ATATTAGAGCTCAAATAAATCCTGGGCGCATA | construct *menF* deletion; SOE primer D | SacI |
| GC251 | ATATTACCATGGAAATTCCAGCCGATGTTACG | cloning *menD* into pIMK2 | NcoI |
| GC252 | ATATTAGTCGACAAAGCTGGCTTTTCTCCACTT | cloning *menD* into pIMK2 | SalI |
| GC253 | ATATTACCATGGAAACGCAAACCAAGCAGTTT | cloning *menF* into pIMK2 | NcoI |
| GC254 | ATATTACCCGGGCAGCACTTGTTCGTGGTTTG | cloning *menF* into pIMK2 | XmaI |
| GC304 | CGAGGACAACCAGTTTTTTTACATGAATAAATTTGAGGCATTAGCCATGTTTTTCTCTC | construct *menA* deletion; SOE primer B |  |
| GC305 | GAGAGAAAAACATGGCTAATGCCTCAAATTTATTCATGTAAAAAAACTGGTTGTCCTCG | construct *menA* deletion; SOE primer C |  |
| GC307 | ATATTAGGTACCATCTACTTGGCGCTGGAAAC | construct *menA* deletion; SOE primer A | KpnI |
| GC308 | ATATTAGAGCTCTGCGATTACGATTTCGGATA | construct *menA* deletion; SOE primer D | SacI |
| GC310 | GCGATTAATAGAAACCATAACTCATTTAGTGACAAAAAATAGTTTATCCACCCTTTTCTCCTCCT | construct *cydAB* deletion; SOE primer B |  |
| GC311 | AGGAGGAGAAAAGGGTGGATAAACTATTTTTTGTCACTAAATGAGTTATGGTTTCTATTAATCGC | construct *cydAB* deletion; SOE primer C |  |
| GC313 | ATATTAGGTACTTGAAGAAGCCGAGAAAGC | construct *cydAB* deletion; SOE primer A | KpnI |
| GC314 | ATATTAGAGCTCCGGAAGCTGCATTTTCTAT | construct *cydAB* deletion; SOE primer D | SacI |
| GC477 | ATATTACCATGGCAGACGAAGGTGGATTAAACG | clone *lmo1757* into pIMK2 | NcoI |
| GC478 | ATATTACCCGGGAAGAACTGTCCTCTCCTGCAT | clone *lmo1757* into pIMK2 | XmaI |
| GC479 | ATATTACCATGGAAAAGGAATATGGAGGTGTGGA | clone *lmo1602* into pIMK2 | NcoI |
| GC480 | ATATTACCCGGGCCATGTTAAATCCCTCCAA | clone *lmo1602* into pIMK2 | XmaI |
| GC481 | ATATTACCATGGGAGTTTTAGAAGGGGAAA | clone *pdhC* into pIMK2 | NcoI |
| GC482 | ATATTACCCGGGTGGAAAATCGCCTACTACCA | clone *pdhC* into pIMK2 | XmaI |
| GC483 | ATATTACCATGGACACAATATATAGTTAGTGGGGGATT | clone *nrdD* into pIMK2 | NcoI |
| GC484 | ATATTACCCGGGCCATTCACACGGTTTTGGAT | clone *nrdD* into pIMK2 | XmaI |
| GC485 | ATATTACCATGGTGTTTATACAAACCAACCAGTAAGGA | clone *lmo2427* into pIMK2 | NcoI |
| GC486 | ATATTACCCGGGCAAAAGCCGTTTTCGACTTC | clone *lmo2427* into pIMK2 | XmaI |
| GC500 | CCCCTTTTTACTTCCCTGCGTCAAGTACCTTTGACACCCGAATCCC | construct *qoxA* deletion; SOE primer B |  |
| GC501 | GGGATTCGGGTGTCAAAGGTACTTGACGCAGGGAAGTAAAAAGGGG | construct *qoxA* deletion; SOE primer C |  |
| GC503 | ATATTAGGTACCGCCAATGCTTCAAATCGAAA | construct *qoxA* deletion; SOE primer A | KpnI |
| GC504 | ATATTAGGTACCACAAGTTGGCCCACATCATT | construct *qoxA* deletion; SOE primer D | KpnI |
| GC505 | ATATTAGGTACCGTCAAAGTAGAAATCTTCAAAACC | construct *lmo1602* deletion; SOE primer A | KpnI |
| GC506 | ATATTAGGTACCCTTCTAAAAATCTGCTCCAA | construct *lmo1602* deletion; SOE primer D | KpnI |
| GC535 | TTACACCTTCAAATCAAGGGAATTTTGGCCAATTAAAACTCATTTTTCTTCCTCCTTCAA | construct *menB* deletion; SOE primer B |  |
| GC536 | TTGAAGGAGGAAGAAAAATGAGTTTTAATTGGCCAAAATTCCCTTGATTTGAAGGTGTAA | construct *menB* deletion; SOE primer C |  |
| GC538 | ATATTAGGTACCAATATGCTAGTAAACGGTCAACATTA | construct *menB* deletion; SOE primer A | KpnI |
| GC539 | ATATTAGAGCTCGGGAATGTCACGCTGTTTG | construct *menB* deletion; SOE primer D | SacI |
